# Post-translational modifications of GlmR integrate metabolic and stress signals to maintain cell envelope homeostasis in *Bacillus subtilis*

**DOI:** 10.1371/journal.pgen.1012096

**Published:** 2026-03-30

**Authors:** Logan B. Suits, Sebastian J. Khan, Dipanwita Bhattacharya, Silviya Dimitrova, Prahathees J. Eswara

**Affiliations:** 1 Department of Molecular Biosciences, University of South Florida, Tampa, Florida, United States of America; 2 Present address: Cell and Developmental Biology Center, National Heart, Lung, and Blood Institute, National Institutes of Health, Bethesda, Maryland, United States of America; 3 Center for Antimicrobial Resistance, University of South Florida, Tampa, Florida, United States of America; Texas A&M University, UNITED STATES OF AMERICA

## Abstract

The metabolic networks of most life forms integrate cost-benefit analysis to properly budget carbon and other essential nutrients. *Bacillus subtilis* is a Gram-positive model bacterium found in diverse ecological niches such as soil, marine environments, and the human gut. As such, *B. subtilis* cells fine-tune metabolic pathways by monitoring signals indicating the presence of nutrients and stressors. A highly conserved protein, GlmR, is a key player in rationing carbon for the production of cell envelope precursors. This function of GlmR can be attributed to its role in cell shape regulation and antibiotic resistance. Given its central position in carbon utilization, GlmR is under post-translational regulation by phosphorylation and UDP-N-acetylglucosamine (UDP-GlcNAc) binding. GlmR is also linked to cyclic-di-AMP (c-di-AMP), a nucleotide second messenger involved in osmotic and cell wall stress response. In this study, we probed the importance of GlmR in cell morphogenesis, c-di-AMP signaling, and investigated the physiological significance of post-translational regulation. Our results reveal that cells lacking *glmR* exhibit: (i) increased susceptibility to tunicamycin, a cell envelope targeting antibiotic; (ii) impaired division site positioning; and (iii) reduced intracellular c-di-AMP concentration. Furthermore, we show that the function of GlmR is fine-tuned by UDP-GlcNAc binding, phosphorylation, and acetylation. Additionally, we provide evidence showing that the recently discovered uridyltransferase activity of GlmR is integral for its function. We show that GlmR is a cell width determinant and propose a model suggesting close cooperation with an actin-like protein, MreB. Overall, our studies highlight the importance of the enzymatic function of GlmR and elucidate the mechanism behind the multiple post-translational means to regulate this crucial protein which is at the crux of carbon flux with an important role in maintaining cell envelope integrity.

## Author summary

Bacteria must integrate feedback from multiple metabolic processes and environmental cues to efficiently allocate carbon to produce essential building blocks such as nucleotides, amino acids, and cell wall precursors to support life. GlmR is one such critical carbon flow regulator in diverse bacterial phyla. In *Bacillus subtilis*, absence of GlmR leads to abnormally large cells that are hypersensitive to cell wall targeting antibiotics. Our data reveals that the generation of cell wall precursors is an important contribution of GlmR, as growth is severely inhibited when its enzymatic function is disrupted. Based on our results, we provide a model that explains the various cell shape phenotypes previously associated with GlmR. As siphoning off too much carbon from other essential processes is detrimental to cell viability, GlmR activity is tightly regulated. In this report, we elucidate the importance of GlmR post-translational modification such as phosphorylation and lysine acetylation in calibrating carbon utilization.

## Introduction

**Fig 1:**
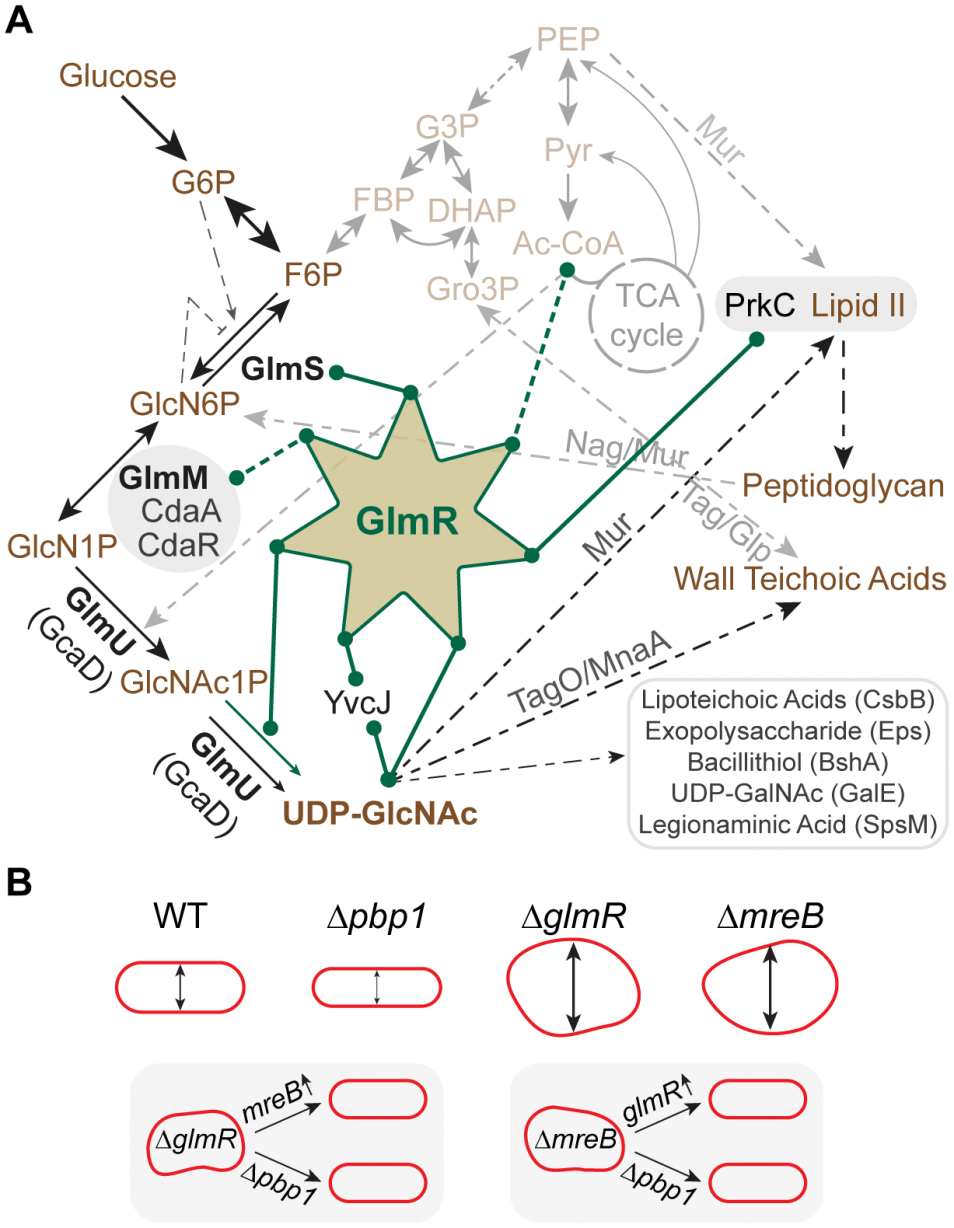
GlmR plays a central role in carbon metabolism and cell shape determination. **(A)** GlmR-centric metabolic pathway showing carbon flux in *B. subtilis*. Abbreviations: G6P, glucose-6-phosphate; F6P, fructose-6-phosphate; FBP, fructose 1,6-bisphosphate; G3P, glyceraldehyde 3-phosphate; DHAP, dihydroxyacetone phosphate; Gro3P, glycerol-3-phosphate; PEP, phosphoenolpyruvate; Pyr, pyruvate; Ac-CoA, acetyl-coenzyme A; TCA, tricarboxylic acid; GlcN6P, glucosamine-6-phosphate; GlcN1P, glucosamine-1-phosphate; GlcNAc1P, N-acetylglucosamine-1-phosphate; UDP-GlcNAc, UDP-N-acetylglucosamine. The green lines connected to GlmR indicate direct (solid line) or indirect (dashed line) regulation and/or functions. Solid and dashed black lines represent single or multiple steps respectively. Gray arrows indicate additional pathways not discussed in detail in this article. Processes/products that utilize UDP-GlcNAc are indicated with enzymes involved listed above the arrows or within parentheses. Enzymes needed to support the uridyltransferase activity (green arrow) of GlmR are shown in bold font. The catalytic activity of GlmR requires two consecutive aspartates at position 38 and 39. GlmR is acetylated at lysine 296 using Ac-CoA directly or indirectly with the help of another protein. GlmR is phosphorylated at threonine 304 by PrkC which is believed to sense Lipid II abundance. GlmR is regulated by UDP-GlcNAc binding which requires arginine 301. The function of GlmR in stimulating GlmS is modulated by another UDP-GlcNAc binding protein YvcJ. The genes *yvcJ* and *glmR* are encoded together. Evidence linking GlmR and the nucleotide second messenger cyclic-di-AMP (c-di-AMP) exists. *cdaA*-*cdaR*-*glmM* genes constitute a highly conserved operon encoding genes that form a tripartite complex which determines the intracellular concentration of c-di-AMP. *glmS* riboswitch-ribozyme activation/inhibition is indicated. Cartoon depiction of cell morphology of *B. subtilis* wild type (WT) and *glmR*, *pbp1* (*ponA*), and *mreB* deletion mutants. Deletion of *pbp1* results in thinner cells while deletion of *mreB* leads to increased cell width when viability is maintained via magnesium supplementation. Cells lacking *glmR* also are abnormally large when grown in gluconeogenic conditions. The cell morphology defects of both Δ*glmR* and Δ*mreB* can be corrected by either deletion of *pbp1* or by overexpression of *mreB* or *glmR* respectively.

To survive and thrive, bacteria must be adept at tuning the metabolic pathways to shuttle carbon for building genetic material, cell envelope, and generating energy. This regulation must happen within seconds to assess, calibrate, and shunt metabolic intermediates to appropriate biosynthetic cycles. Post-translational regulation of metabolic proteins and the use of second messengers greatly aid in achieving this instantaneous response [1,2]. *Bacillus subtilis* is a common soil bacterium generally considered as the model for Gram-positive organisms [3]. Many bacteria including *B. subtilis* occupy and adapt to diverse ecological niches including the human gut [4] and marine environments [5]. Therefore, *B. subtilis* cells must skillfully tackle a multitude of challenges such as varying levels of nutrients, osmotic shifts, and other types of stressors. To achieve this, they must tightly ration carbon for different essential processes such as DNA replication, cell envelope synthesis, cell division, as well as energy production to power these tasks [6,7]. Additionally, carbon partitioning decisions should be made in conjunction with the assessment of nutrient availability and environmental threats such as those that weaken the cell envelope [8]. GlmR (formerly YvcK) is a key metabolic factor involved in carbon utilization (Fig 1A), specifically in the production of precursors needed for peptidoglycan (PG) synthesis and other cell envelope components [8–10]. In this report, we examine the physiological impacts of GlmR, its absence, and the various regulatory means available to calibrate its function.

### Antibiotic resistance

As GlmR plays a key role in the accumulation of PG precursors, its presence becomes crucial in the presence of cell wall stress. Specifically, it has been noted that cells lacking *glmR* exhibit increased sensitivity to several cell wall targeting antibiotics [11,12]. This includes different classes of antibiotics that inhibit cell wall synthesis such as bacitracin, vancomycin, moenomycin, cefuroxime, and oxacillin. Thus, GlmR is a critical antibiotic resistance factor.

### Cell shape

It is known that GlmR becomes essential in conditions requiring gluconeogenesis [13]. Cells lacking *glmR* grown in the absence of glucose exhibit abnormal morphology (Fig 1B). Therefore, Δ*glmR* phenotypes can be suppressed by either glucose or magnesium supplementation [12,13]. While glucose would allow glycolysis and negate the need for gluconeogenesis, the effect of magnesium is likely multifactorial. This is because magnesium supplementation may inhibit PG hydrolases [14–16], deplete intracellular PG precursor levels [17], and facilitate osmoregulation [18]. Besides chemical supplementation, deletion of *pbp1* (*ponA*), the gene encoding class A bifunctional penicillin binding protein (PBP1), also abrogates the cell morphology and viability defects of cells lacking *glmR* (Fig 1B) [19]. Additionally, overproduction of MreB, an actin-like cytoskeletal protein [20], also restores rod shape in Δ*glmR* strain [19]. Conversely, overexpression of *glmR* or deletion of *pbp1* corrects the cell morphology defects of a strain harboring *mreB* deletion [19,21]. Balanced activities of MreB and PBP1 have been recognized to govern the width of the cell [22,23]. Given the reciprocal phenotypes of *mreB* and *glmR* mutants, it is possible GlmR is also a cell width determining factor. In this report we present evidence in support of this notion.

### Cell wall precursor synthesis

The first step to commit carbon for the PG precursor pathway is taken by GlmS, an enzyme involved in the de novo synthesis of GlcN6P (key to abbreviations are provided in Fig 1 legend). *glmS* transcript abundance is self-regulated through autocleavage by a GlcN6P-responsive riboswitch-ribozyme [24]. This ribozyme activity is hindered by glucose and G6P [25,26]. Intriguingly, changes in intracellular pH and magnesium concentration may influence the activity of this ribozyme [27,28]. Alternatively, peptidoglycan can be recycled into GlcN6P to supplement this pathway [29]. Subsequently the enzymatic actions of GlmM and GlmU (GcaD) help generate the essential cell wall precursor, UDP-GlcNAc. In *B. subtilis*, GlmR directly interacts with GlmS and stimulates its enzymatic activity [12,30,31]. Moreover, it was shown recently that GlmR of *B. subtilis* and other species are in fact uridyltransferase enzymes capable of producing UDP-GlcNAc [31]. Therefore, GlmR plays a central role in carbon utilization by influencing the accumulation of UDP-GlcNAc (Fig 1A). Consequently, the flow of carbon to make PG precursors can be calibrated by regulating GlmR.

### Nucleotide second messenger signaling

Past studies have linked mutations that bypass the need for *glmR* to increased expression of *glmM* and/or *glmS* [12]. This genetic locus harbors the highly conserved *cdaA*-*cdaR*-*glmM* operon (Fig 1A) [32]. CdaA is the major cyclic-di-AMP (c-di-AMP) synthase and is regulated by CdaR and GlmM in *B. subtilis* and other organisms [33–37]. Intriguingly, disruption of c-di-AMP phosphodiesterase genes *pgpH* (*yqfF*) or *gdpP* (*yybT*) also alleviate *glmR* phenotypes [12,13]. Yet, the mechanism behind this remains unclear.

### Post-translational regulation

GlmR binds UDP-GlcNAc, the product of its catalytic activity (Fig 1A). When UDP-GlcNAc level is in excess, this ligand binding is thought to weaken the GlmR-mediated stimulation of GlmS to establish a negative feedback loop [30,38]. Additionally, YvcJ, a RapZ-like protein encoded from the gene immediately upstream of *glmR*, also binds UDP-GlcNAc and interacts specifically with UDP-GlcNAc bound GlmR [30]. Besides ligand binding, GlmR (T304 residue) is also subject to phosphoregulation by PrkC [11], a S/T kinase speculated to regulate cell wall homeostasis by sensing the level of Lipid II precursors [39–41]. Furthermore, a proteomics study identified that GlmR (K296 amino acid) is acetylated [42]. Metabolic intermediates such as acetyl-CoA and its derivative acetyl-phosphate are known to serve as donors of acetyl group for lysine acetylation [43,44].

In this study, we sought to investigate the new and unresolved questions regarding GlmR in antibiotic resistance, cell morphogenesis, and c-di-AMP signaling. In addition, we probed the importance of enzymatic activity, UDP-GlcNAc binding, phosphorylation, and acetylation of GlmR. Our results reveal that GlmR aids in resisting tunicamycin, an antibiotic that targets wall teichoic acids (WTA) biosynthesis selectively and the PG pathway at higher concentrations. We observe that cells lacking GlmR exhibit reproducibly larger cell width compared to the wild type (WT) control even when irregular cell morphology is corrected with chemical supplementation. Reciprocally, we see a reduction in cell width with *glmR* overexpression. We notice that division site positioning is impaired when *glmR* is deleted. We also note that c-di-AMP level is affected by GlmR in a manner dependent on CdaA. Our experiments demonstrate that the uridyltransferase activity of GlmR is essential for its cellular function, as the catalytically inactive mutant is unable to complement the Δ*glmR* phenotypes. Finally, we show that UDP-GlcNAc binding, phosphorylation, and lysine acetylation fine-tune GlmR activity. We provide a model based on our results to explain the role of GlmR in reversing the phenotypes of cells lacking actin-like proteins MreB and Mbl. In sum, our results shed light on the multi-level regulation of GlmR and its crucial role in cell morphogenesis and antibiotic resistance.

## Results

### GlmR is important for tunicamycin resistance

**Fig 2:**
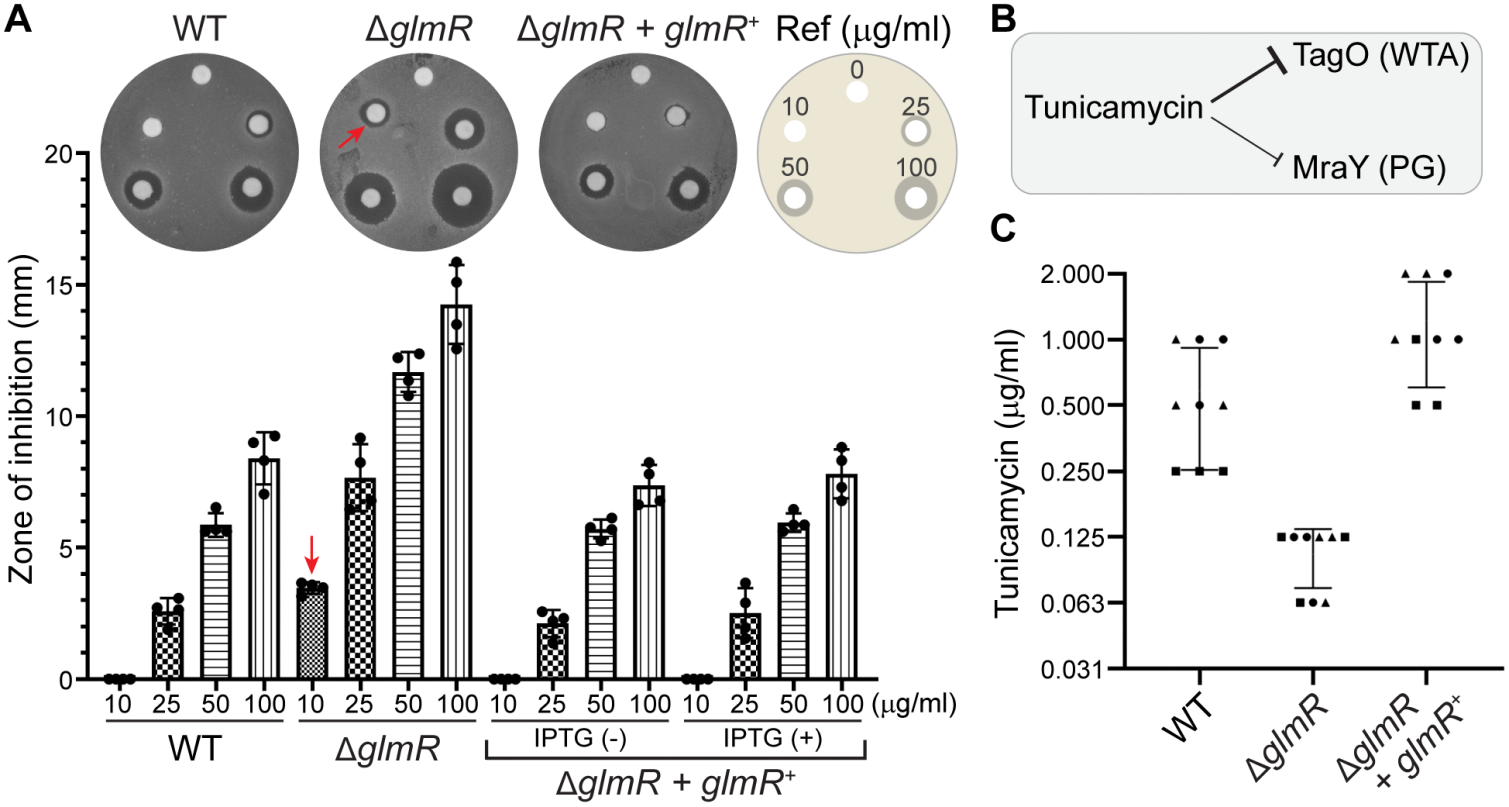
Cells lacking *glmR* exhibit increased tunicamycin sensitivity. **(A)** Disks containing 5 µl of either 0, 10, 25, 50, or 100 µg/ml tunicamycin were placed (as shown in reference; Ref) on lawns made of WT (PY79), ∆*glmR* (SK35), and ∆*glmR* complemented with IPTG-inducible *glmR* (SK56). Absence of GlmR leads to increased tunicamycin susceptibility, as evidenced by increased zone of inhibition (ZOI) compared to WT and the complementation strain. Quantification of the ZOI (minus the 6.5 mm disk) Also plotted are data for SK56 strain grown in the absence (leaky expression) or presence (1 mM) of IPTG. Average of four replicates is shown, and standard deviation is displayed as error bars. The red arrow indicates the lowest concentration where ZOI is observed for ∆*glmR* but not WT. **(B)** Enzymes targeted by tunicamycin. TagO, an enzyme critical for synthesizing wall teichoic acids (WTA) is selectively inhibited at lower concentration range while MraY involved in peptidoglycan (PG) synthesis is also inhibited at higher concentrations. **(C)** Minimum inhibitory concentration (MIC) of tunicamycin was assessed for WT (PY79), ∆*glmR* (SK35), and Δ*glmR* harboring inducible *glmR* (SK56) all grown in LB containing 1 mM IPTG. The triangles, circles, and squares correspond to experiments performed on different days. Error bar represents standard deviation.

Given the essentiality of UDP-GlcNAc in WTA production (Fig 1A) [45], we reasoned that cells lacking GlmR may display increased susceptibility to antibiotics that target the WTA pathway, such as tunicamycin (Fig 2). The nucleoside antibiotic, tunicamycin (an analog of UDP-GlcNAc) is known to inhibit glycosyltransferases [46]. More specifically, the first step of WTA biosynthesis mediated by TagO is selectively inhibited by tunicamycin at a lower concentration range, while at a significantly higher range (>100x concentration in *S. aureus* [47]; > 50 µg/ml in *B. subtilis* [48,49]) hinders the function of MraY involved in the PG synthesis pathway (Fig 2B) [49–51]. To test whether there are any changes in tunicamycin susceptibility between WT and cells lacking *glmR*, we conducted a disk diffusion assay encompassing a range of concentrations. Briefly, sterile disks laced with 0, 10, 25, 50, and 100 µg/ml of tunicamycin were placed on the lawns of either WT or Δ*glmR* strains (Fig 2A). The WT strain did not exhibit any noticeable sensitivity at 10 µg/ml and we observed a small but measurable zone of inhibition (ZOI) at 25 µg/ml. Remarkably, consistent with our prediction, we observed a larger ZOI for Δ*glmR* compared to WT control at all concentration ranges including 10 µg/ml, the lowest concentration tested (Fig 2A; see red arrows). The increased susceptibility of Δ*glmR* was abolished with *glmR* complementation, even in the absence of inducer likely due to leaky expression. Next, we determined the minimum inhibitory concentration (MIC) of tunicamycin in lysogeny broth (LB). Based on our assay, the MIC for WT is ~ 0.5 µg/ml (Fig 2C). This is well within the range of previous reports that show a MIC anywhere between 0.15 µg/ml to 24 µg/ml [49,51–53], depending on the growth media and parental *B. subtilis* strain backgrounds. In contrast, we find the MIC for Δ*glmR* to be ~ 0.125 µg/ml which suggests that cells lacking *glmR* are nearly 4-fold more sensitive to tunicamycin. The resistance is restored and ~2-fold enhanced by ectopic complementation of *glmR* (MIC ~ 1 µg/ml). As GlmR plays a key role in supplying UDP-GlcNAc (Fig 1A) for both TagO and MraY enzymes, it is conceivable that the biogenesis of both WTA and PG (the two most important cell envelope components [54]) are dysregulated in the absence of GlmR. Thus, cells lacking *glmR* are ill-equipped to counter antibiotics that target these pathways. Based on our results, we add tunicamycin to yet another class of antibiotics for which sensitivity is elevated in the absence of GlmR.

### GlmR is required for cell width maintenance

**Fig 3:**
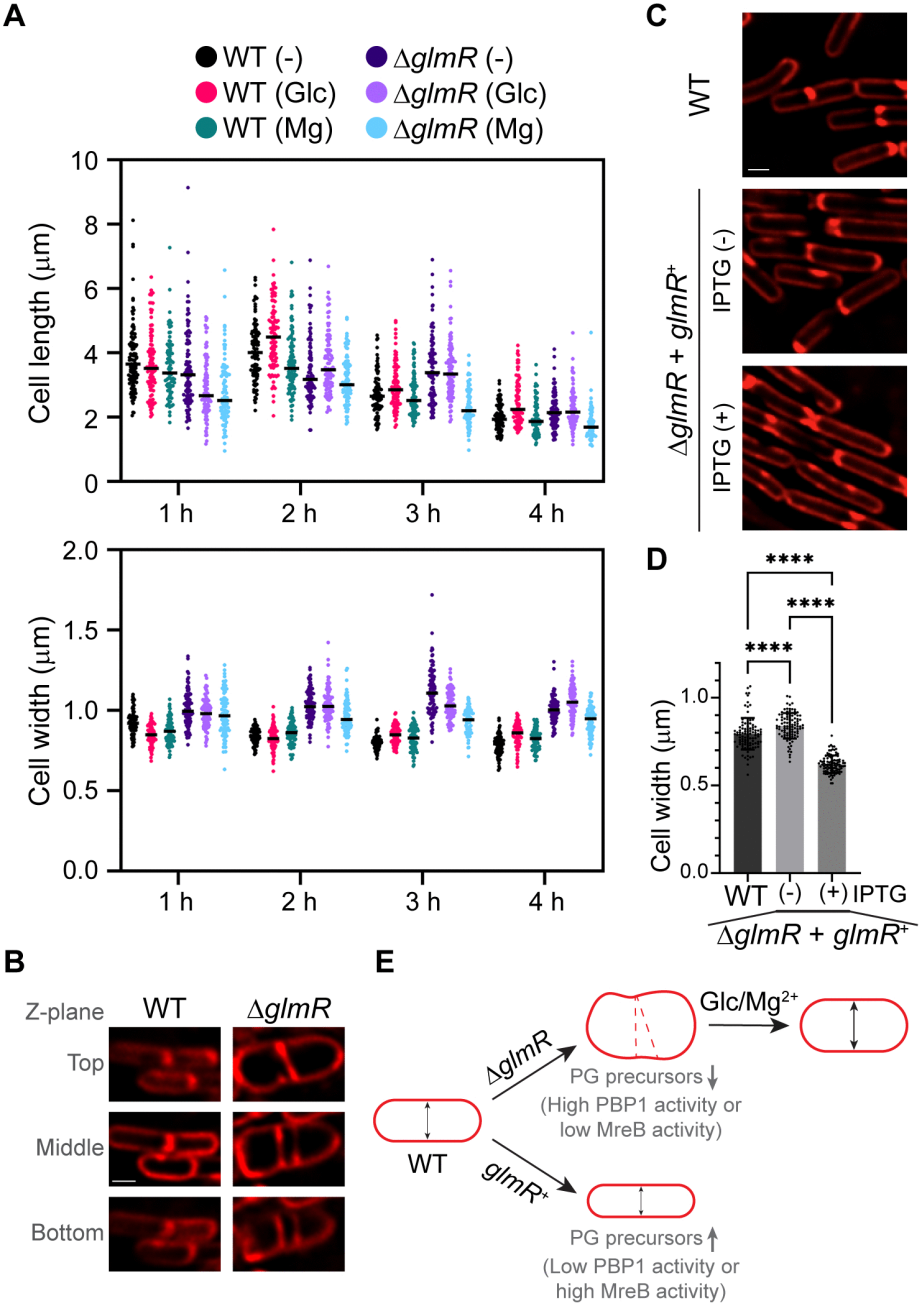
GlmR is a cell width determinant. **(A)** Cell length and width quantification of WT (PY79) and ∆*glmR* (RB176) strains grown in LB in the absence or presence of D-glucose (1%) or magnesium (25 mM MgCl_2_) supplementation grown for up to 4 hours and imaged at every hour. Representative micrographs are shown in S1 Fig. Individual data points and corresponding average (black line) of indicated time points post-supplementation or mock are shown (n = 100). **(B)** Micrographs showing normal and abnormal septation in WT (PY79) and ∆*glmR* (SK35) respectively. See **S1** **Video** to view all frames of ∆*glmR*. Additional examples are indicated in S1 Fig with yellow arrows. Red, FM 4-64 membrane dye. Scale bar, 1 µm. **(C)** Representative micrographs showing WT (PY79) and ∆*glmR glmR*^*+*^ (SK56) uninduced or induced with 1 mM IPTG. Scale bar, 1 µm. **(D)** Cell width quantification of panel C data (n = 100). Cells were measured using FIJI. Statistical significance was assessed through one-way ANOVA with Tukey’s correction; **** = p < 0.0001. **(E)** Graphical summary depicting key observations. Although glucose or magnesium supplementation abrogates the abnormal morphology of ∆*glmR* cells, the average cell width is consistently larger than WT control at all time points. Additionally, we note that overexpression of *glmR* leads to reduced cell width and that deletion of *glmR* impairs division site positioning indicated as red dashed lines.

Given that *glmR* and *mreB* mutants phenocopy each other (Fig 1B), we tested whether GlmR is also involved in cell width maintenance similar to MreB. As mentioned earlier, it is known that glucose or magnesium supplementation supports Δ*glmR* cell viability in conditions where GlmR is otherwise essential [13]. Thus, we examined the morphology of WT and Δ*glmR* cells using fluorescence microscopy with or without glucose and magnesium supplementation in LB. We quantified the cell morphology changes for 4 h to cover both exponential growth phase and transition to stationary phase (Fig 3A). As expected [13], Δ*glmR* cells exhibited abnormal cell bulging compared to WT control in the absence of any supplementation (S1 Fig). We also observed increased incidence of aberrantly proximal and irregular septation in the Δ*glmR* strain with nearly 40% of septa in the field of view (n = 100) when compared to WT (<5%; n = 100) (Fig 3B and S1 Video). Thus, when GlmR is absent, the positioning of cytokinetic machinery is impaired, as evidenced by irregular septation.

The cell length changes over time were consistent with previous observations for WT (Fig 3A) [55,56]. A similar trend was seen for the cells lacking GlmR. Δ*glmR* cells at the 3 h timepoint were longer than the WT control, which was noted previously [38]. However, by the 4 h mark this difference was diminished. In contrast to cell length, the cell width difference between WT and Δ*glmR* was more striking – with the latter being consistently wider. Magnesium addition corrects the abnormal Δ*glmR* cell morphology as reported previously [13]. We find that magnesium supplementation leads to shorter cell length at nearly all time points regardless of the strain type. This specific effect of magnesium on *B. subtilis* cell length has been reported previously [57]. Nonetheless, the cell width is consistently larger than WT. Although the aberrant cell morphology in the absence of GlmR is known, we expected glucose supplementation to allow glycolysis (as GlmR function is believed to be prominent only during gluconeogenesis [19]) and restore cell width similar to that of WT. However, our results reveal that Δ*glmR* cells are wider than WT at all timepoints even in the presence of glucose. Likewise, the post-exponential phase cell widths at 4 h were also greater than the WT control with or without supplementation. Reciprocally, unlike deletion, overexpression of *glmR* which presumably increases the abundance of PG precursors, leads to decreased cell width (Fig 3CD). Based on these results, we infer that GlmR is a key cell width determinant (Fig 3E), possibly through assisting PBP1 localization [19] and/or by feeding enough PG precursors to balance MreB (rod complex)/PBP1 consumption (Fig 1B).

### GlmR influences the intracellular c-di-AMP level

**Fig 4:**
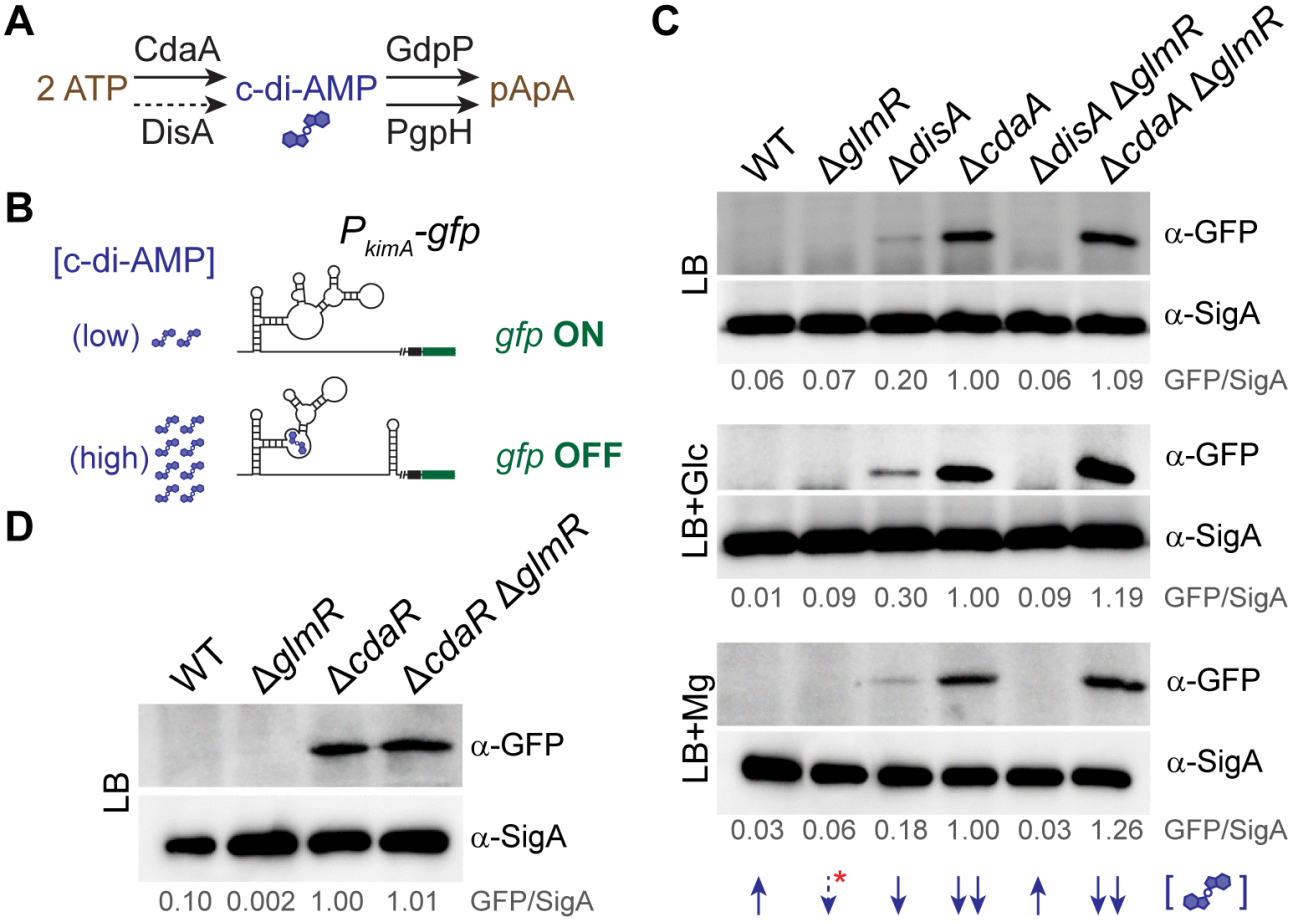
GlmR influences the CdaA-mediated synthesis of c-di-AMP. **(A)** Enzymes involved in the c-di-AMP synthesis and turnover. CdaA and DisA are the major and minor c-di-AMP synthases respectively. *B. subtilis* encodes a third c-di-AMP synthase, CdaS, that is activated specifically during sporulation (not indicated here). GdpP and PgpH are phosphodiesterases involved in the conversion of c-di-AMP to phosphoadenylyl-adenosine (pApA). Solid lines and dashed lines indicate the major and minor enzymes respectively. **(B)** C-di-AMP riboswitch reporter of *kimA* was used to generate a GFP-based reporter. Transcription of *gfp* is dependent on low intracellular c-di-AMP concentration. **(C)** Representative western blots of *P*_*kimA*_*-gfp* reporter in WT (SK94), ∆*glmR* (SK113), ∆*disA* (SK109), ∆*cdaA* (SK110), ∆*disA* ∆*glmR* (SK111), and ∆*cdaA* ∆*glmR* (SK112) backgrounds. All strains were grown in LB without or with supplementation of D-glucose (1%) or MgCl_2_ (25 mM) and harvested 2 hours after induction. Antibodies against GFP and SigA (loading control) were used to monitor the changes in *P*_*kimA*_*-gfp* reporter activity. Blue arrows indicate inferred changes in c-di-AMP levels relative to WT (↑). Red asterisk is based on results shown in S2E Fig. **(D)** Immunoblot of WT (SK94), ∆*glmR* (SK113), ∆*cdaR* (SK118), and ∆*glmR ∆cdaR* (SK119) probed with GFP or SigA antisera. The GFP/SigA ratio for all lanes relative to either ∆*cdaA* or ∆*cdaR* are shown.

In vegetatively growing *B. subtilis*, c-di-AMP synthesis occurs mainly through CdaA and DisA, which are the major and minor synthases respectively (Fig 4A) [12,36,58,59]. It is known that cells lacking both are nonviable [60,61], thus highlighting the vital role of c-di-AMP signaling. Given that mutations in the *cdaA* locus or deletion of c-di-AMP hydrolases allow Δ*glmR* phenotype suppression [12,13,62], we wished to probe the intracellular c-di-AMP level in cells lacking *glmR*. For this, we engineered a reporter in which *gfp* expression was controlled by the c-di-AMP sensing riboswitch of *kimA* (*ydaO*) promoter [63–65]. Briefly, when the intracellular level of c-di-AMP drops, *gfp* will be transcribed and when c-di-AMP level is high *gfp* expression will halt (Fig 4B). Therefore, based on our design, lack of GFP indicates presence of c-di-AMP and vice versa. Similarly designed reporters have been successfully utilized for this purpose by other groups [66,67]. We introduced this reporter in the WT background as well as in strains harboring individual or combinatorial deletions of *glmR* and *cdaA*. Previous reports have found that relative to WT, deletion of *disA* leads to a modest decrease in intracellular c-di-AMP concentration [67–70]. As our probe lacks the ability to distinguish high vs. very high levels of c-di-AMP, to enhance the sensitivity of our reporter, we also included *disA* deletion. In WT and Δ*glmR*, we did not detect a GFP band suggesting these cells maintain relatively high intracellular c-di-AMP concentration (Fig 4C). As expected, cells lacking *disA* produced a faint band while the *cdaA* knockout strain produced an intense band. Interestingly, in the strain harboring deletions of both *disA* and *glmR* genes, the faint GFP band was no longer present (Fig 4C; compare Δ*disA* and Δ*disA* Δ*glmR* lanes). This trend was consistent and reproducible even when the respective cultures were grown in the presence of glucose or magnesium. We infer this to mean that lack of GlmR, when DisA is absent, leads to stimulation of c-di-AMP production. We do not observe any noticeable changes in *cdaA glmR* double deletion as the GFP band intensity corresponding to this strain resembles that of *cdaA* knockout. Thus, the increased c-di-AMP levels we notice in Δ*disA* Δ*glmR* strain appears to be dependent on CdaA. Next, we tested whether this stimulation requires CdaR, a known modulator of CdaA activity [36,61]. Deletion of both *cdaR* and *glmR* also produced a prominent GFP band similar to Δ*cdaA* and Δ*glmR* (Fig 4D). Our results suggest that both CdaA and CdaR are responsible for the elevated c-di-AMP levels in the Δ*disA* Δ*glmR* strain. Therefore, it appears that the absence of GlmR in Δ*disA* background leads to stimulation of c-di-AMP production through the major c-di-AMP synthase CdaA. However, as our results are derived from cells lacking either DisA or CdaA, they do not reveal the true influence that GlmR may exert on c-di-AMP level. Therefore, we used a commercial competitive Enzyme-Linked Immunosorbent Assay (ELISA) kit to directly address this (S2E Fig). We ensured the functionality of the assay using WT and Δ*cdaA* strains as our controls. Intriguingly, we did not see an increase in c-di-AMP level in the absence of GlmR, in fact we see a modest decrease in this second messenger concentration. This result is however in line with our *P*_*kimA*_*-gfp* reporter which suggested that both WT and Δ*glmR* strains contain relatively high intracellular c-di-AMP concentration. On the contrary, we see that overproduction of GlmR leads to a small but reproducibly elevated c-di-AMP level compared to WT. This is consistent with a recent report that revealed a link between high c-di-AMP concentration and reduced cell width and vice versa [67], as we also note similar cell width changes for GlmR (Fig 3E). Taken together, our results inform that GlmR level influences the intracellular concentration of the c-di-AMP second messenger in a manner dependent on CdaA.

Inspired by our results, we probed whether alterations in c-di-AMP levels influence the Δ*glmR* cell morphology. We find that deletion of both *cdaA* and *glmR* results in a stunning reversal of aberrant cell morphology (S2A Fig). However, as discussed in S1 Text, we attribute this to a polar effect that may synthetically elevate the expression of the downstream gene *glmM* of the *cdaA*-*cdaR*-*glmM* operon (S2BC Fig) [71,72]. Notably, suppression of Δ*glmR* phenotypes by increased levels of GlmM has been reported previously [12,62]. Regardless, we note that deletion of *cdaA* or *cdaR* individually or in combination with Δ*glmR* results in a significant drop in intracellular c-di-AMP level (Fig 4CD) - yet they all maintain normal cell shape (S2A Fig). Based on this, we conclude that reduced intracellular c-di-AMP concentration is not detrimental to cells when the cell envelope is not compromised or stressed.

We also tested whether other c-di-AMP signaling related enzymes indicated in Fig 4A influence the ∆*glmR* phenotypes. For this, we investigated the double deletion phenotypes of a strain devoid of *glmR* and the gene encoding the minor c-di-AMP synthase DisA. We also included the strains lacking GlmR and one of the two c-di-AMP phosphodiesterases GdpP or PgpH. Our experiments show that additional deletion of *gdpP* or *pgpH*, which would elevate the c-di-AMP level by limiting its turnover, is unable to correct the abnormal ∆*glmR* cell shape defect (S2AD Fig). So is the case with Δ*disA* Δ*glmR*, which also increases intracellular c-di-AMP concentration (Fig 4C). However, they all appear to correct the growth phenotypes to a varying degree (S3 Fig). Our interpretations of these results and associated phenotypes are discussed in S1 Text.

### The enzymatic activity of GlmR is integral for its function

**Fig 5:**
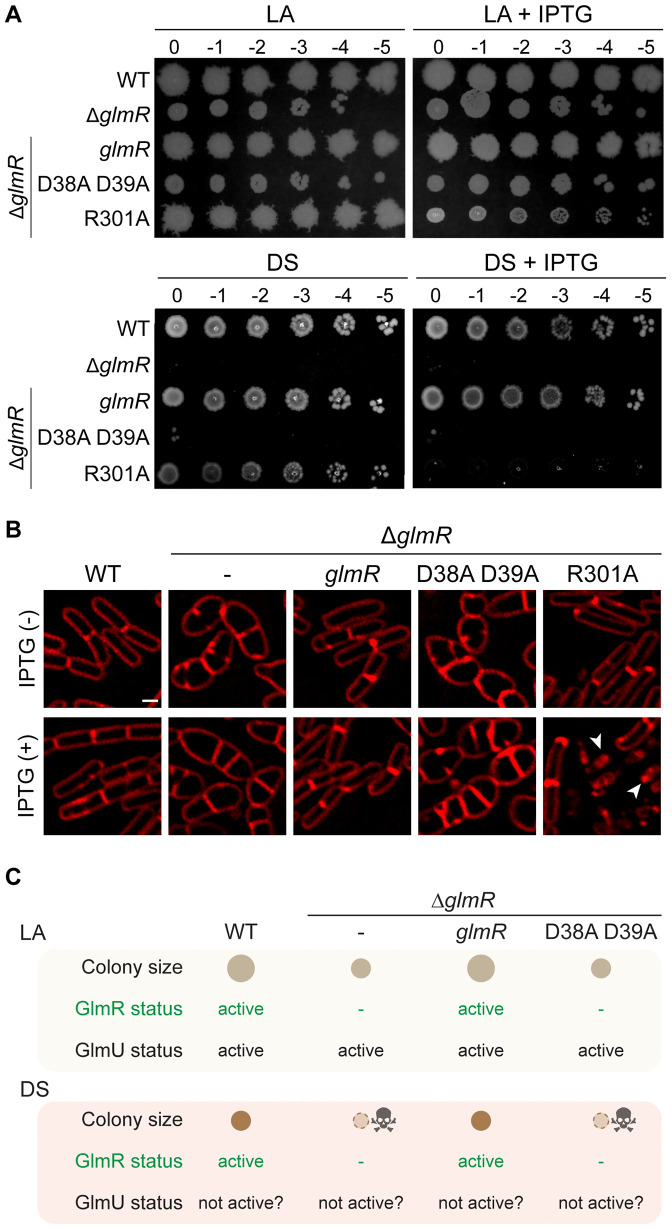
Enzymatic activity of GlmR is essential for its function. **(A)** Serial dilutions of WT (PY79), ∆*glmR* (RB176) and ∆*glmR* complemented with IPTG-inducible *glmR* (SK56)*,* D38A D39A *glmR* mutant that is catalytically inactive (BLS95), or R301A mutant which is deficient in UDP-GlcNAc binding (SK141). Representative pictures of LA and DS plates incubated overnight at 37 °C are shown. Inducer plates contain IPTG (1 mM) when indicated. **(B)** Micrographs of strains listed in panel A grown in LB without or with (1 mM) IPTG. Cells are labeled with FM 4-64 membrane dye (red). Scale bar, 1 μm. Arrow heads point to lysed cells. **(C)** Tabulated summary of data shown in panel A indicating the functionality of GlmR and speculated activity of GlmU in the indicated strain backgrounds and growth media.

GlmR was recently shown to be an uridyltransferase [31]. Therefore, we aimed to probe the physiological relevance of the enzymatic function of GlmR. To examine this, we generated strains in which Δ*glmR* is complemented with an inducible copy of either unmutated *glmR* or *glmR* variant harboring catalytic site mutations (D38A D39A; [31]). We then tested the growth of these strains on LB agar (LA) and Difco starch (DS) plates (Fig 5A). On LA (which contains magnesium [57]), when compared to WT, Δ*glmR* cells grew, albeit poorly. In contrast, on DS, while the WT strain grew well, cells lacking *glmR* exhibited severe growth inhibition and appear to be nearly non-viable. This medium is similar to Mueller-Hinton (MH) which is prohibitive for supporting the growth of Δ*glmR* strain [12]. However, we are unsure why Δ*glmR* cells are unable to utilize starch to support glycolysis. It is possible defective WTA may hinder the secretion of amylase needed for starch degradation [73]. Nevertheless, both the small colony phenotype on LA and lethality on DS can be reversed by *glmR* complementation. Thus, DS growth medium serves as a useful tool to study the functionality of GlmR mutants.

Interestingly, we find that the D38A D39A mutant is unable to complement the Δ*glmR* phenotypes on LA and DS (Fig 5A). Cell morphology analysis through microscopy reveals that D38A D39A mutant is not capable of correcting the cell shape abnormality commonly seen in Δ*glmR* strain (Fig 5B). We used functional His-tagged GlmR to ensure that the D38A D39A mutant is stably produced (S4A Fig). Our results reveal that the catalytic activity of GlmR is required for complementation and therefore for its normal cellular function. GlmU is the primary (essential) enzyme responsible for UDP-GlcNAc production (Fig 1A). As depicted in Fig 5C, combined action of GlmU and GlmR is likely needed to support WT-like growth on LA. Thus, we see poor growth without the enzymatic function of GlmR. On the other hand, on DS medium, our results indicate that GlmR becomes the sole enzyme responsible for UDP-GlcNAc production. This notion is further strengthened by our finding that the catalytically inert mutant is unable to support the growth of Δ*glmR* strain.

Next, we tested whether UDP-GlcNAc binding by GlmR affects growth by making use of the R301A mutant that lacks the ability to bind to this ligand. As previously reported [12,38], R301A complements Δ*glmR* phenotypes. However, we note this happens on LA and DS only in the absence of inducer due to leaky expression - unlike the catalytically inert mutant (Fig 5A). However, in the presence of inducer, the phenotype is starkly different. We noticed a decrease in colony diameter on LA + IPTG and no growth on DS + IPTG. Thus, overexpression of R301A closely mimics Δ*glmR* phenotype in these conditions. Upon probing these cells through microscopy, we find that the cells harboring an inducible copy of R301A mutant are more similar to WT in the absence of inducer and prone to lysis in the presence of inducer (Fig 5B; see arrowheads). UDP-GlcNAc binding is proposed to promote GlmR-YvcJ interaction and thereby prevent GlmR-mediated stimulation of GlmS (Fig 1A) [30]. Therefore, it is possible that the R301A mutant may continuously stimulate GlmS (or possess enhanced enzymatic activity) which is detrimental to cell viability. Although the differential effect on YvcJ phenotype for this mutant has been noted [30], the toxic effect upon overexpression was not. This data highlights the regulatory potential of UDP-GlcNAc ligand – which is the product of the enzymatic activity of GlmR.

### GlmR phosphorylation may negatively affect UDP-GlcNAc binding

GlmR is a substrate of the S/T kinase PrkC and is phosphorylated at T304 [11]. Therefore, we tested the ability of T304A (phosphoablative) and T304E (phosphomimetic) versions of GlmR to complement Δ*glmR* phenotypes. Our results indicate that both mutants function equivalently to unmutated *glmR* and complement all *glmR* knockout phenotypes: growth on LA and DS (S5A Fig) and restoration of cell morphology (S5B Fig). We also note that similar to *glmR* overexpression (Fig 3CD), overproduction of phosphomimetic or phosphoablative versions of GlmR results in cell width reduction (S5C Fig). Thus, phosphorylation does not appear to significantly influence GlmR function. This observation is consistent with previous reports [11,12]. Upon closer inspection, we do notice a modest but reproducibly smaller colony size for T304A phosphoablative mutant when compared to the GlmR control and T304E variant (S5D Fig). It has been noted that GlmR phosphomutants have differential effects on bacitracin sensitivity, correction of aberrant cell shape of Δ*mreB*, and PBP1 localization [11]. Thus, we aimed to further investigate the specific purpose of GlmR phosphoregulation.

**Fig 6:**
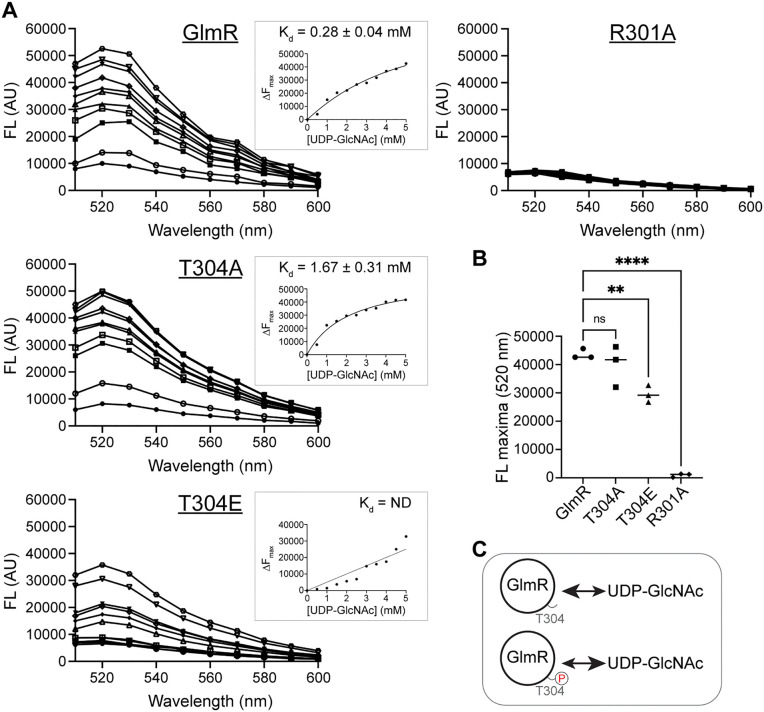
Phosphorylation of GlmR may weaken UDP-GlcNAc binding. **(A)** The binding of UDP-GlcNAc to purified WT GlmR, GlmR T304A, GlmR T304E, or GlmR R301A were monitored using fluorescence spectroscopy. Shown are the representative fluorescence spectra of FITC-labeled GlmR or mutants (5 µM) were incubated without (●) or with UDP-GlcNAc at 0.5 mM (○), 1.0 mM (■), 1.5 mM (□), 2.0 mM (▲), 2.5 mM (△), 3.0 mM (◆), 3.5 mM (◇), 4.0 mM (▼), 4.5 mM (▽) and 5.0 mM (⬡) concentrations. Inset, binding constants (*K*_*d*_) were calculated by plotting the difference in fluorescence intensities (Δ*F*_*max*_) at different ligand concentrations when saturation was achieved. ND, not determined. **(B)** The fluorescence maxima of GlmR (●), T304A (■), T304E (▲), and R301A (◆) are plotted; n = 3, ** and **** indicate p < 0.0084 and 0.0001, respectively. **(C)** Inference model based on the results shown in panel A indicating that phosphoregulation of residue T304 may alter the kinetics of UDP-GlcNAc ligand binding.

Given the proximity of T304 phosphosite to R301, the residue important for UDP-GlcNAc binding, GlmR phosphorylation may influence ligand recognition [38]. Therefore, we tested this possibility with purified GlmR. For this, we used FITC-labeled protein (WT GlmR or mutants) and monitored the fluorescence change associated with ligand (UDP-GlcNAc) binding to study the binding kinetics [74]. The saturation of change in fluorescence (Δ*F*) associated with UDP-GlcNAc titration allowed us to estimate the dissociation constant (*K*_*d*_) for GlmR as 0.28 ± 0.04 mM (Fig 6A; see inset). This value is close to 0.41 mM predicted previously [38], highlighting the reliability of this approach. In contrast, for our negative control R301A mutant, we did not notice any change in fluorescence. This confirms that R301A mutant is unable to bind UDP-GlcNAc as established previously [38]. For T304A mutant, our estimated *K*_*d*_ value is 1.67 ± 0.31 mM, which is in a similar range to GlmR control. On the contrary, for T304E mutant, Δ*F* saturation was not achieved even with 5 mM UDP-GlcNAc, which precluded us from calculating the *K*_*d*_ value. We infer this result to mean that UDP-GlcNAc binding is possibly weaker and/or more transient when GlmR is in a phosphomimetic state (Fig 6B). Overall, our results indicate that GlmR phosphorylation may render UDP-GlcNAc binding less favorable (Fig 6C). As PrkC is involved in the regulation of cell envelope synthesis and is important for resisting cell wall targeting antibiotics [39–41], GlmR phosphorylation may be used by cells to temporarily and reversibly override self-inhibition by UDP-GlcNAc accumulation to accelerate PG precursor production and strengthen the cell envelope.

### GlmR function is likely affected by acetylation

**Fig 7:**
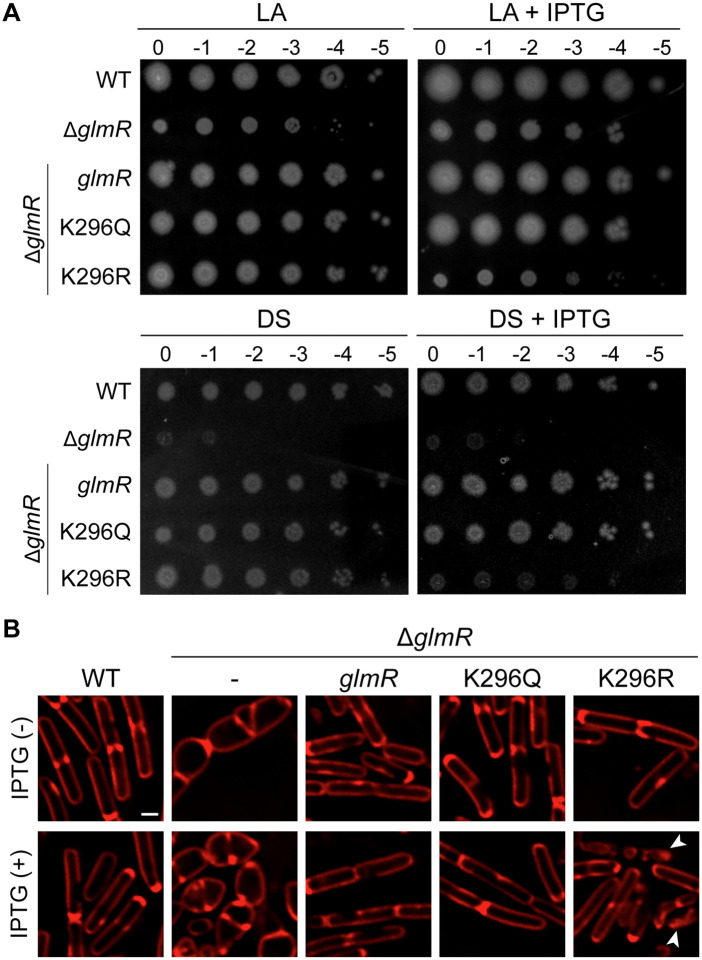
GlmR function is moderated by acetylation. **(A)** Spot titers of WT (PY79), ∆*glmR* (RB176) and ∆*glmR* complemented with IPTG-inducible *glmR* (SK29)*,* K296Q *glmR* mutant to mimic acetylation (SK107), or K296R *glmR* mutant to mimic unacetylated status (SK108). Representative plate pictures of the above-mentioned strains grown in LA or DS medium at 37 °C overnight are shown. Inducer plates contain IPTG (1 mM). **(B)** Fluorescence micrographs of strains listed in panel A grown in LB in the absence or presence IPTG (1 mM). Cell membrane is stained with FM 4-64 dye (red). Scale bar, 1 μm. Arrowheads indicate cell lysis.

**Fig 8:**
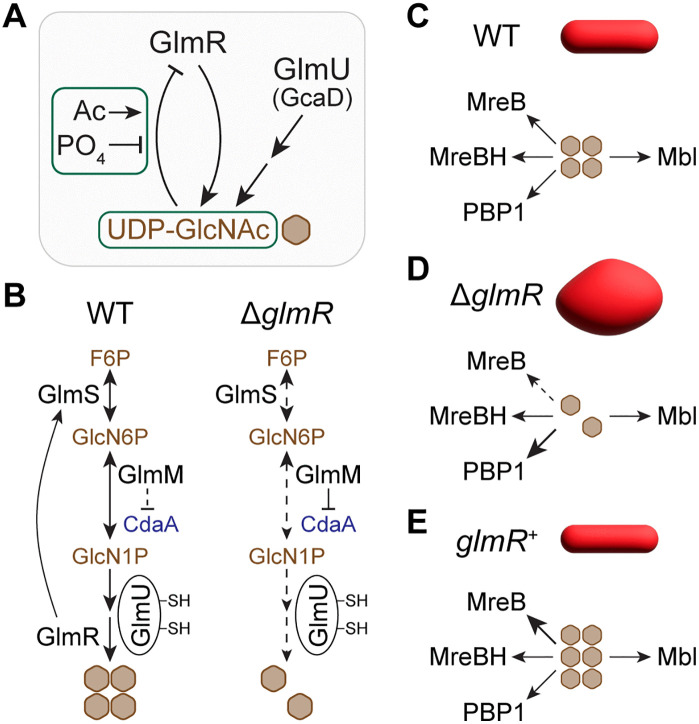
Graphical summary and illustration of proposed models. **(A)** In *B. subtilis,* GlmU is the main bifunctional essential enzyme that generates UDP-GlcNAc. In this report, we show that the uridyltransferase activity of GlmR is also critical to supplement UDP-GlcNAc synthesis. We also reveal that UDP-GlcNAc binding inhibits the catalytic activity of GlmR. While lysine acetylation (Ac) promotes this inhibition, phosphorylation appears to weaken UDP-GlcNAc binding and thus may prevent self-inhibition. **(B)** De novo amino sugar biosynthetic pathway involved in UDP-GlcNAc production. We predict that GlmM inhibition of CdaA is moderate when the forward reaction is effective such as when GlmR is present. In the absence of GlmR, we speculate carbon flow in this pathway is decreased. This signal is transduced through increased inhibition of CdaA by GlmM. This decreased CdaA activity contributes to a modest drop in intracellular c-di-AMP concentration. SH labels on GlmU indicate two redox-sensitive cysteines that negatively affect its acetyltransferase activity in non-reducing conditions. **(C)** Speculative model depicting the main UDP-GlcNAc utilization pathways. The actin-like proteins MreB, MreBH, and Mbl assist with RodA/class B PBP machinery; PBP1 is a class A bifunctional enzyme. MreB/MreBH control cell width. Mbl is believed to monitor cell elongation. PBP1 is considered a general-purpose cell wall repair enzyme. Proper deployment of these four pathways is critical for cell width regulation. **(D)** In the absence of GlmR, we predict less accumulation of UDP-GlcNAc. We believe that this specifically impairs MreB pathway (see S6 Fig). **(E)** We suspect that overproduction of GlmR leads to elevated UDP-GlcNAc level which is specifically consumed through the MreB pathway. Hence, the decrease in cell width we observe in cells overexpressing *glmR* (Fig 3). Arrows: dashed and increased thickness are used to indicate weak and strong flow/consumption/interaction respectively.

In addition to phosphorylation, GlmR (also known as MgfK) is subject to another post-translational modification through lysine acetylation at position 296 [42]. Several other metabolic enzymes shown in Fig 1A, including GlmS, GlmM, GlmU, and YvcJ are also acetylated [42,43,75,76]. Acetylation is known to regulate catalysis, influence protein structure, and alter partner preference between proteins [77]. Therefore, we aimed to study whether lysine acetylation influences GlmR function. To investigate this, we mutated K296 to either glutamine to mimic acetylation or arginine to resemble an unacetylated residue as previously described [42]. As shown in Fig 7A, K296Q acetyl-mimetic mutant was able to complement Δ*glmR* phenotypes in LA and DS similar to the *glmR* control. In contrast, while K296R acetyl-ablative mutant was able to complement in the absence of inducer, overexpression resulted in significant growth inhibition (Fig 7A; see + IPTG plates). Microscopy analysis revealed that K296Q acetyl-mimetic mutant corrected the abnormal Δ*glmR* cell morphology, similar to the unmutated *glmR* control (Fig 7B). On the other hand, K296R acetyl-ablative mutant prevented the cell bulging phenotype of Δ*glmR* only in the absence of inducer. In the presence of inducer, we observed increased cell lysis (Fig 7B; see arrowheads). Thus, it appears that acetylation may also moderate the activity of GlmR by possibly hindering UDP-GlcNAc binding as these observations resemble the phenotypes of R301A variant (Fig 5). Taking the proximity of the sites important for UDP-GlcNAc binding (R301) and phosphorylation (T304) and our results into account, we propose that acetylation of GlmR (K296) likely negatively affects the flow of carbon to the PG precursor pathway. Conversely, we believe deacetylation and phosphorylation may promote UDP-GlcNAc synthesis (Fig 8A).

## Discussion

### Antibiotic resistance

The enzymatic role of GlmR in drawing carbon for UDP-GlcNAc synthesis is now evident (Fig 8AB). As such, cells devoid of *glmR* are incapable of mounting a proper response to host immune factors and cell wall targeting antibiotics [11,12,31,78–80]. Given that UDP-GlcNAc is also an essential component of the WTA pathway, we show that cells lacking *glmR* are highly sensitive to tunicamycin (Fig 2), a dual-function antibiotic that preferentially targets WTA at lower concentrations and PG biosynthesis at higher concentrations.

### Cell morphogenesis

The push and pull of PBP1 and MreB (rod complex) activities determine the cell width of *B. subtilis* cells [21–23]. The similarities between *glmR* and *mreB* phenotypes hinted at an analogous role for GlmR (Fig 1B). We now confirm that GlmR is a key cell width determinant (Fig 3E). Besides MreB, *B. subtilis* encodes two additional MreB-like proteins Mbl and MreBH (Figs 8C and S6A) [20,81]. Although *mreB* and *mbl* are essential and *mreBH* is not, the essentiality could be reversed in certain growth or genetic conditions [81]. While MreB and MreBH play a role in the same pathway to achieve cell width control, Mbl is involved in an independent but parallel pathway to facilitate cell elongation [82,83]. In a high-throughput CRISPR-based analysis, it was recently found that *mbl* can be rendered non-essential in the absence of *glmR* [84]. This is in direct contrast to MreB, where *glmR* overexpression supports the viability of cells lacking *mreB* (Fig 1B) [19]. Thus, it may be reasoned that GlmR differentially modulates MreB, Mbl, and PBP1 functions. However, based on the enzymatic function of GlmR, we speculate that the differences in synthetic essentiality are likely due to the different rate of PG precursor consumption and/or PG hydrolysis in these mutants, which can be supported by either *glmR* deletion (for Δ*mbl*) or overexpression (for Δ*mreB*). In the absence of GlmR, we expect a reduction in UDP-GlcNAc level (Fig 8B), which disproportionately affects the MreB pathway of elongasome machinery (Fig 8D). This is supported by the finding that only overexpression of *mreB* restores the cell morphology defects of cells lacking *glmR* - but not *mbl* or *mreBH* [19]. Upon GlmR overproduction, we predict that UDP-GlcNAc level would be elevated and specifically favors MreB pathway, thus leading to decreased cell width (Fig 8E).

Another genetic condition that leads to Δ*glmR* cell shape restoration is deletion of *pbp1* (Fig 1B) [19]. Eliminating the consumption of UDP-GlcNAc by PBP1 likely frees up this precursor for MreB. Additionally, *pbp1* deletion triggers alternative sigma factor SigI activation and consequently upregulation of *mreBH* ensues (S6AB Fig) [85]. This likely reinforces UDP-GlcNAc utilization by the collaborative MreB/MreBH pathway [82]. This may also explain why deletion of *pbp1* or SigI activation suppresses the aberrant cell shape defects of both *mreB* and *mbl* knockout strains [81,86]. As depicted in S6A Fig, the morphology of cells lacking *mbl* is quite distinct from Δ*mreB* mutant [87–89]. In the extended discussion (see S1 Text), we use our working model to explain this peculiar Δ*mbl* phenotype, why deletion of *glmR* renders *mbl* non-essential, and why glucose and magnesium supplementation fail to restore the cell width of Δ*glmR* cells to resemble WT (Fig 3E). Overall, we believe that GlmR dictates the cell width by supplying UDP-GlcNAc to bolster MreB activity. Of note, we find that Δ*glmR* cells exhibit increased sensitivity to tunicamycin an antibiotic that targets the WTA pathway (Fig 2). It is shown that the MreB-containing rod complex is specifically inhibited by tunicamycin and that PBP1 activity becomes more important in these cells [49]. Thus, the cell bulging that happens when WTA production is inhibited by either deletion of *tagO* [90] or tunicamycin treatment [91] is possibly due to hindered MreB activity and increased PBP1 function (Fig 8D). We do note that our rather simplistic model does not take other factors with known role in cell shape regulation into account [83,92–95]. Thus, further model refinement is warranted when more information regarding GlmR comes to light.

### Cytokinesis

We observed that the positioning of cytokinetic machinery is impaired when GlmR is absent (Fig 3B). The underlying mechanism for this is unclear at the moment. Specifically, how division site positioning systems are bypassed needs to be investigated. It is possible that altered carbon metabolism, turgor pressure, cell morphology, and/or membrane fluidity may lead to aberrant cytokinesis [7,96–99]. Alternatively, it could be due to decreased WTA composition as cells lacking *tagO* also exhibit aberrant septation [90].

### Connections to c-di-AMP

In *B. subtilis* and other organisms, GlmM inhibits CdaA (Fig 8B) [33–37]. GlmR increases the substrate availability for GlmM by stimulating GlmS. GlmR also contributes to the removal of the product made by GlmM (GlcN1P) to generate UDP-GlcNAc. Thus, it is conceivable that both actions of GlmR would promote the enzymatic function of GlmM to favor the forward reaction. We suspect that this may in turn result in weaker inhibition of CdaA activity, which allows WT cells to maintain a relatively high intracellular c-di-AMP concentration (Fig 4). This argument is strengthened by our result showing GlmR overproduction leads to increased c-di-AMP level (S2E Fig). Conversely, absence of GlmR would result in GlmM idling longer (or performing the reverse reaction) which may promote GlmM dependent CdaA inhibition (Fig 8B) and consequently lead to decreased c-di-AMP concentration. Thus, this nucleotide second messenger may be used by the cells to broadcast the status of GlmM function. Instead (or in addition), changes in turgor pressure due to a weakened cell wall [67], which we observe in Δ*glmR* strain manifested by abnormal cell morphology, could lead to altered enzymatic activity of CdaA. Therefore, the cytoplasmic regulation of CdaA by GlmM combined with the extra-cytoplasmic regulation of CdaA by CdaR (Fig 4C), both detecting flawed cell wall synthesis in the absence of GlmR function may negatively affect c-di-AMP level [61,67,100]. We find that increased expression of *glmM* in Δ*glmR* is sufficient to support WT-like growth and cell morphology even when *cdaA* is deleted (S2C Fig). Thus, we can postulate that the reduction of intracellular c-di-AMP level is not deleterious by itself and may signify when the cell envelope needs reinforcement such as when the PG precursor pathway is inhibited or in the presence of cell wall stressors.

One of the main functions of c-di-AMP is to regulate potassium influx/efflux for osmoregulation [18,101–109]. We elaborate on the possible implications in the extended discussion provided in S1 Text. Additionally, secreted c-di-AMP [69] and/or stringent response activation [110–114] may influence metabolic pathways in the absence of GlmR. Thus, further experiments are needed to test these possibilities.

### Enzymatic activity

GlmR and its homologs possess uridyltransferase activity to synthesize UDP-GlcNAc (Fig 8A) [31]. We show that the catalytic function of GlmR is: (i) essential for growth on DS, (ii) required to support WT-like growth on LA, and (iii) necessary to correct Δ*glmR* cell morphology defects (Fig 5). The *B. subtilis* GlmU (GcaD; an essential protein) has both acetyltransferase [115] and uridyltransferase [116] functions to generate N-acetyl glucosamine-1-phosphate (GlcNAc-1P) and UDP-GlcNAc respectively (Figs 1A and 8AB). Based on our results, we can infer that on DS, the uridyltransferase activity of GlmU is disabled and cells exclusively depend on GlmR (Fig 5C). Could GlmR perform both functions of GlmU? This possibility was ruled out in an in vitro experiment with purified *L. monocytogenes* GlmR [31]. Whether in vivo acetylation of GlmR (Fig 7) allows for it to be an acetyltransferase remains to be seen. The key lysine residue is mutated in both acetyl-mimetic (K296Q) and acetyl-ablative (K296R) mutants of GlmR, yet they complement Δ*glmR* phenotypes. Thus, perhaps one of the acetylated partners of GlmR such as YvcJ may serve as an acetyl donor. Intriguingly, the morphogenetic protein MreB (discussed above) is also acetylated at three different residues and the acetyl-ablative mutation of one of the lysines impairs cell morphology control [42]. Additional experiments are necessary to investigate these models.

In *Mycobacterium tuberculosis*, phosphorylation of GlmU by an S/T kinase was found to negatively affect the acetyltransferase activity but not uridyltransferase activity [117]. Thus, one of the two functions of GlmU could be specifically deactivated by post-translational modification. It was found that *glmU* gene expression is downregulated during stringent response [118,119]. The production of stringent response alarmone, (p)ppGpp, can be elicited by a c-di-AMP binding protein, DarB [112]. As we observed GlmR-mediated fluctuations in c-di-AMP concentration (Figs 4 and S2E), perhaps on DS medium *glmU* transcription is repressed by stringent response activation. Pyruvate kinase is another metabolic enzyme with link to stringent response [120], which is differentially autoregulated in glycolytic vs gluconeogenic conditions [121]. Thus, it is possible that GlmU activity is affected similarly. Additionally, conditions such as high magnesium and low pH hinder the acetyltransferase activity of *B. subtilis* GlmU [115]. Intriguingly, alteration in c-di-AMP levels is also known to modulate intracellular magnesium concentration [18]. Moreover, it has been found that GlmU is regulated by two cysteine residues that are involved in disulfide bond formation and therefore responsive to intracellular redox status (Fig 8B) [115,122]. Specifically, acetyltransferase activity was found to be diminished in non-reducing conditions. It is therefore plausible that the intracellular conditions of Δ*glmR* mutant in DS growth medium are not optimal for one or both enzymatic function(s) of GlmU. Intriguingly, UDP-GlcNAc is also essential for bacillithiol production (Fig 1A). Bacillithiol is an important agent involved in redox stress response as well as a resistance factor for cell wall targeting antibiotics [123]. Therefore, it is tempting to speculate that in the absence of GlmR cells are unable to properly respond to oxidative stress. However, overexpression of *glmS*/*glmM* (S2BC Fig) appears to fully restore GlmU activity perhaps by alleviating oxidative stress. Alternatively, the cause of death of Δ*glmR* cells and failure to complement by enzymatically inert mutant could be indirect. For example, this mutant may fail to stimulate GlmS and draw carbon for GlmU to execute its function (Fig 8B). Experiments to test these speculations are underway.

### Phosphorylation

Prior to the knowledge of its uridyltransferase activity, GlmR was shown to bind UDP-GlcNAc. The mutants defective in binding UDP-GlcNAc did not exhibit any noticeable phenotype [12,38]. However, we show that the binding of UDP-GlcNAc ligand moderates GlmR function (Fig 8A) as overproduction of UDP-GlcNAc binding deficient variant (R301A) is toxic (Fig 6AB). Similarly, phosphoregulation of GlmR and its physiological importance have been investigated previously [11,12]. GlmR mutants either mimicking phosphorylated or unphosphorylated state support growth. PrkC, the kinase responsible for phosphorylating GlmR, is known to sense cell wall stress and/or the changes in the levels of PG precursors and fine-tune growth rate accordingly [39–41,124]. Thus, it is possible that phosphorylation of GlmR may serve to prioritize carbon for strengthening the cell envelope through boosting UDP-GlcNAc level. Subsequently, the removal of phosphate by PrpC phosphatase may lower the function back to basal level but not completely off - this would explain why phosphoablative mutant is also able to complement. Results of our in vitro experiments suggest that phosphorylation may hinder but not abolish UDP-GlcNAc binding (Fig 8A). Intriguingly, while overexpression of the mutant deficient in recognizing UDP-GlcNAc (R301A) is toxic, phosphomimetic mutant (T304E) where UDP-GlcNAc binding appears to be more transient is not. However, GlmR is subject to multiple levels of regulation within the cell that cannot be incorporated with purified proteins. As only phosphomimetic version of GlmR supports rod shape maintenance in Δ*mreB* [11], we could envision a scenario where phosphorylation increases UDP-GlcNAc synthesis and support Mbl/MreBH pathway to counteract the action of PBP1 (Fig 8C). On the contrary, phosphoablative mutant is unable to sufficiently elevate the UDP-GlcNAc level in Δ*mreB* strain background to support rod shape maintenance. Alternatively, as MreB and PBP1 form a complex [21] and GlmR assists in PBP1 localization [11,19], perhaps GlmR phosphorylation alters the partner preference. Further investigation is required to test these hypotheses.

### Acetylation

Similar to phosphorylation, acetylation of lysine residues serves as another reversible post-translational regulatory mechanism [77]. A handful of proteomics studies have identified acetylated proteins in *B. subtilis* [42,43,75,76]. GlmR is found to be acetylated along with several other proteins including GlmS, GlmM, GlmU, and YvcJ discussed in this report (Fig 1A). Therefore, we investigated whether acetylation influences GlmR function. Our results revealed that GlmR acetyl-mimetic (K296Q) mutant was equally efficient as WT copy in complementing *glmR* phenotypes. However, acetyl-ablative GlmR mutant (K296R) was toxic upon overexpression similar to the mutant (R301A) that is unable to bind UDP-GlcNAc. Based on our results, it appears that acetylation of lysine 296 of GlmR may serve to negatively regulate its enzymatic activity (Fig 8A). Interestingly, the equivalent residue is naturally glutamine in *Bacillus halodurans* and based on the crystal structure (PDB ID: 2O2Z) it does not appear to be involved in the coordination of its ligand, NAD in this case [38]. However, this loop-like region is not clearly resolved in the crystal structure and is possibly dynamic. In *Staphylococcus aureus* and *Listeria monocytogenes*, the corresponding residue appears to be aspartate and glutamate respectively [31]. Our heterologous complementation experiment suggests that *S. aureus* GlmR (*Sa* GlmR) exhibits uridyltransferase activity in vivo as it complements *B. subtilis* Δ*glmR* strain (S4C Fig). This is consistent with a previous finding [31]. Intriguingly, we see that the overproduction phenotype of *Sa* GlmR resembles that of *B. subtilis* K296R acetyl-ablative mutant (Fig 7A). Nonetheless, other proximal lysine residues are present in GlmR homologs, and may be acetylated in the corresponding host organism. Therefore, acetylation is likely an additional post-translational mechanism that fine-tunes the function of GlmR [125–127]. Besides acetylation, GlmR appears to be also subject to succinylation and malonylation [76]. Thus, multiple modes of regulation may exist.

### Conservation of GlmR and its potential as drug target

Our findings about *B. subtilis* GlmR are likely broadly applicable to its homologs in other organisms which include several clinically relevant pathogens. GlmR is highly conserved in diverse bacterial lineages and in some archaea [13,30]. However, except for Gram-positive bacteria not much is known about the function of GlmR-like proteins in other organisms. In *M. tuberculosis* (CuvA/Rv1422), it is found to accumulate at sites of active cell wall synthesis and contribute to virulence [78]. It is also found to be phosphorylated by an S/T kinase in this organism [128]. *L. monocytogenes* GlmR is important for survival within the host during infection, and it is also phosphorylated by an S/T kinase [79]. In *S. aureus*, *glmR* is an essential gene [129,130]. However, another study indicates that *glmR* mutant is temperature sensitive [131]. A link between GlmR, S/T kinase, and CdaA appears to exist in *S. aureus* as well [132]. *Enterococcus faecalis* GlmR phenotype differs from that of its *B. subtilis* counterpart [80]. Nevertheless, it binds to UDP-GlcNAc and plays a role in antibiotic resistance. GlmR homologs of the above-mentioned organisms are enzymatically active and produce UDP-GlcNAc [31,80]. The specific function of *E. coli* homolog (YbhK) is yet to be characterized. However, *yhbK* of *E. coli* was found to complement *glmR* in *B. subtilis* – thus it may play a similar enzymatic function in its host organism as well [13]. Disruption of *yhbK* is associated with increased tolerance to the cell wall targeting antibiotic ampicillin [133]. Intriguingly, GlmR in *Salmonella enterica* is regulated via glycosylation, specifically with GlcNAc addition to an arginine [134]. Thus, additional experiments are needed to uncover the full scope of the regulatory modes of GlmR and other related pathways. Given its crucial role in antibiotic resistance and pathogenesis, inhibitors of GlmR enzyme could be developed as novel therapeutics.

### Summary

*B. subtilis* is an excellent model to investigate fundamental biological pathways. Additionally, it is also a widely used bacterium in various biotechnology industries [3,135]. Thus, deeper understanding of metabolic processes would propel the fields of both basic and applied biology. In this report, we show that GlmR is a central metabolic enzyme which is regulated by multiple means. More specifically our results suggest that post-translational regulation of GlmR may aid in decisions involving carbon prioritization. This role becomes vital in times of cell envelope stress. Therefore, unsurprisingly, cells lacking *glmR* are highly susceptible to cell envelope targeting antibiotics. As antibiotic resistance is a growing concern worldwide, key metabolic pathways and proteins such as GlmR could serve as potential therapeutic targets [122,136–139].

## Materials and methods

### Strain construction

*B. subtilis* strains utilized in this study are derivatives of PY79 [140]. Specific details regarding strain construction can be found in S1 Text. All strains and oligonucleotides referenced are listed in Tables A and B in S1 Text respectively. The knockout strains were obtained from the Bacillus Genetic Stock Center [141]. Plasmids pDG1662 [142], pDR111 (David Rudner), pDR244 [141,143], and pBS2EXylRP_*xylA*_ (ECE741; [144]) were used to create strains for this study. pET28a vector was used for purifying recombinant *B. subtilis* GlmR and its mutants from *E. coli*. QuikChange kit (Agilent) was used for site-directed mutagenesis. All plasmids generated were verified through Sanger or whole plasmid sequencing (Azenta). All *B. subtilis* chromosomal gene deletions and insertions were confirmed via PCR and other standard techniques.

### Media used and spot titer assay

Overnight cultures grown at 30 °C in lysogeny broth (LB Miller; Fisher Scientific) were serially diluted up to 10^-5^ and 1 µl of culture was plated on LB agar (LA; Fisher Scientific) or BD Difco Starch agar (DS) plates containing either 0 or 1 mM IPTG. Plates were imaged after overnight incubation at 37 °C.

### Antibiotic susceptibility testing

Zone of inhibition (ZOI) assays were completed after overnight cultures of the indicated *B. subtilis* strains were grown in LB at 30 °C. Cultures were standardized to an OD_600_ of 0.1 and 100 µl was spread using sterile glass beads on LA plates. Plates were allowed to dry with their lid off for 30 minutes in a biosafety cabinet. Plates with sterile filter paper disks laced with 5 µl of 0, 10, 25, 50, or 100 µg/ml tunicamycin (Sigma) were incubated overnight at 37 °C. ZOI diameter quantification was performed via FIJI [145]. The standard diameter of the filter paper disks (6.5 mm) was subtracted from all ZOI measurements.

### Minimum inhibitory concentration (MIC) assessment

Overnight cultures of strains grown at 30 °C in LB were diluted to an OD_600_ of 0.01 in 100 µl LB supplemented with 1 mM IPTG. Tunicamycin antibiotic was added to the top row of a 96-well plate and serially diluted down the plate in 2-fold increments. Strains were grown overnight in a 96-well plate at 37 °C in a shaking incubator. The MIC concentration was estimated based on the well with lowest antibiotic concentration that had no growth.

### Western blot

Immunoblot analysis of indicated strains were completed after overnight cultures of *B. subtilis* strains grown in LB at 30 °C were diluted to an OD_600_ of 0.05 in 10 ml of LB. When indicated, cultures were supplemented with a final concentration of 25 mM MgCl_2_, 1% glucose, or 1 mM IPTG. Cultures were grown to an OD_600_ of ~1, standardized to an OD_600_ of exactly 1 before centrifugation and resuspension in protoplast buffer containing 0.5 M sucrose, 20 mM MgCl_2_, 10 mM KH_2_PO_4_, and 0.1 mg/ml lysozyme. Samples were incubated at 37 °C for 30 min and then prepared for SDS-PAGE. After electrophoresis, the samples were transferred onto a nitrocellulose membrane and subsequently probed with appropriate antibodies.

### C-di-AMP enzyme-linked immunosorbent assay (ELISA)

Overnight cultures grown at 30 °C in LB were diluted to an OD_600_ of 0.1 in LB supplemented with 1 mM IPTG. The freshly inoculated strains were grown to an OD_600_ of approximately 1.0 and standardized. A culture aliquot of 1 ml was centrifuged and resuspended in 0.5 ml of protoplast buffer containing 0.5 M sucrose, 20 mM MgCl_2_, 10 mM KH_2_PO_4_, and 0.1 mg/ml lysozyme. Samples were incubated at 37 °C for 30 min and then diluted 50-fold in sterile water to lyse cells. Immediately after this step, ELISA assay was performed according to the manufacturer instructions provided in the kit (Cayman Chemicals). This kit was a kind gift from the laboratory of Dr. Wenqi Yu (USF). All samples were measured in triplicates and read at OD_450_ on a Tecan Infinite 200 PRO plate reader. Analysis was completed in Microsoft Excel and GraphPad Prism 10.

### Microscopy

Overnight cultures of *B. subtilis* strains grown at 30 °C in LB were diluted to an OD_600_ of 0.05 in 10 ml LB. If noted, cultures were made to a final concentration of 25 mM MgCl_2_, 1% glucose, or 1 mM IPTG. For the time course microscopy, cultures were grown for the duration indicated. For all other experiments, cultures were grown to an OD_600_ of 1 at 37 °C. Sample preparation and microscopy techniques were completed as previously described [146]. DeltaVision Elite high-resolution deconvolution fluorescence microscope equipped with Photometrics CoolSnap HQ2 camera was used for imaging. Manufacturer-provided SoftWorx software was used for image processing.

### Quantification and statistics

Analysis of ZOI, colony size, and measurements of cell length/width were performed using FIJI [145]. Statistical analysis was conducted using GraphPad Prism version 10.4.1. Ordinary one-way ANOVA with multiple comparisons was used with Tukey’s post-correction.

### Biochemical analysis

For labeling WT and mutant forms of GlmR, the purified proteins were labeled with fluorescein isothiocyanate (FITC) as previously reported [74]. Briefly, 50 µM of each WT-GlmR and mutants were incubated with FITC (250 µM) in 50 mM phosphate buffer (pH 8.0) for 5 h on ice. The reaction was stopped by adding 5 mM Tris-HCl (pH 8.0). Sephadex G25 fine column (Cytiva) was used to separate the free FITC from the labeled proteins. The concentrations of FITC-bound GlmR and mutants were determined by absorbance at 495 nm. The concentrations of the proteins were measured using Bradford assay. The stoichiometry of labeling FITC per GlmR and mutant constructs was found to be approximately 0.6. The dissociation constant (*K*_*d*_) was determined by titrating UDP-GlcNAc ligand (*L*) and measuring the change in FITC-labeled protein fluorescence (Δ*F*). The following single-site binding equation was used for *K*_*d*_ estimation: Δ*F* = (Δ*F*_*max*_· [*L*])/ (*K*_*d*_ + [*L*]).

## Supporting information

S1 Text Supplemental Results and Discussion Topics. Rescue of ΔglmR phenotypes by additional deletion of cdaA is likely due to polar effect. Deletion of gdpP, pgpH, or disA enhances the growth of ΔglmR mutant. Role of GlmR in cell morphogenesis. Potential role for potassium. Supplemental Methods. Table A: Strains used in this study. Table B: Primers used in this study. III. Supplemental.
(DOCX)

S1 Fig GlmR is important for correct cell shape and septation. Representative micrographs of WT (PY79) and ∆*glmR* (RB176) strains grown in LB in the absence or presence of D-glucose (1%) or magnesium (25 mM MgCl_2_) supplementation, imaged hourly for four hours. Yellow arrows indicate examples of abnormal septation.
(TIF)

S2 Fig Deletion of *cdaA* rescues *∆glmR* phenotype likely due to polar effect. (A) Fluorescence micrographs of membrane-stained (FM 4–64, red): WT (PY79), ∆*glmR* (RB176), ∆*disA* (SK97), ∆*disA ∆glmR* (SK102), ∆*cdaA* (SK97), ∆*cdaA ∆glmR* (SK101) ∆*cdaR* (SK130), or ∆*cdaR ∆glmR* (SK131). Scale bar, 1 μm. (B) Genetic locus of *cdaA*-*cdaR*-*glmM* operon. Genes *sigW-rsiW* are located upstream of this operon while *glmS* is present immediately downstream in the *B. subtilis* genome. Red asterisks indicate the position of mutations commonly found in the ∆*glmR* suppressors that allow increased transcription of *cdaA*-*cdaR*-*glmM* genes and/or *glmS*. The terminator downstream of this *cdaA* operon is weaker (depicted with shorter symbol) resulting in read-through transcription of *glmS*. *glmS* has its own promoter followed by a riboswitch-ribozyme [71,72]. Replacement of *cdaA* or *cdaR* with an antibiotic resistance cassette introduces additional promoter (shown in red) and removal of the cassette replacing *cdaA* (∆cdaA*; markerless) brings *glmM* closer to its native promoter, thus potentially result in stronger expression. (C) Fluorescence micrographs of membrane-stained (FM 4–64, red): WT (PY79), *∆glmR* (RB176), ∆glmR ∆cdaA* (SK138), and ∆glmR ∆cdaA* with inducible *glmM*^*+*^ (BLS67). When indicated, 0 and 1 mM IPTG was used in IPTG (-) and (+) conditions respectively. Scale bar, 1 μm. (D) Representative micrographs of WT (PY79), *∆glmR* (RB176), ∆*gdpP* (SK98), ∆*pgpH* (SK99), *∆glmR ∆gdpP* (SK103), and ∆*glmR ∆pgpH* (SK104). Red, FM 4–64 membrane stain. Scale bar, 1 µm. (E) ELISA-based intracellular c-di-AMP concentration estimation of WT (PY79), ∆*glmR* (SK35), ∆*glmR* complemented with inducible *glmR* (SK56), and ∆*cdaA* (SK130). Filled black circles represent technical replicates; error bars represent standard deviation.
(TIF)

S3 Fig Single deletions of genes related to c-di-AMP. (A) Serial dilutions of WT (PY79), *∆glmR* (SK35), *∆glmR glmS*^*+*^ (BLS84) on LA, LA + 3% xylose, DS, or DS + 3% xylose. (B) Serial dilutions of WT (PY79), *∆glmR* (RB176), *∆glmR ∆cdaA* (SK101), ∆glmR ∆cdaA* (SK138), and ∆glmR ∆cdaA* glmM^*+*^ (BLS67) on LA, LA + 1 mM IPTG, DS, or DS + 1 mM IPTG. (C) Spot titer assay of WT (PY79), ∆*glmR* (SK35), ∆*cdaA* (SK96), ∆cdaA* (SK137), ∆*gdpP* (SK98), and ∆*pgpH* (SK99) on LA and DS. (D) Growth of serially-diluted culture aliquots of WT (PY79), ∆*glmR* (RB176), ∆*glmR* ∆*gdpP* (SK103), and ∆*glmR* ∆*pgpH* (SK104) on LA and DS plates. (E) Spot titer assay showing growth of WT (PY79), *∆glmR* (RB176), *∆disA* (SK97), and *∆glmR ∆disA* (SK102) on LA and DS plates.
(TIF)

S4 Fig Complementation assessment of *B. subtilis* GlmR mutants and *S. aureus* GlmR. (A) Serial dilutions of WT (PY79), *∆glmR* (SK35), *∆glmR glmR*^*+*^ (SK56), *∆glmR glmR-6his*^*+*^ (BLS101), and *∆glmR glmR-D38A-D39A-6his*^*+*^ (BLS102) on LA, LA + 1 mM IPTG, DS, or DS + 1 mM IPTG. (B) Representative western blot of ∆*glmR glmR-6his*^*+*^ (BLS101) and ∆*glmR glmR-D38A-D39A-6his*^*+*^ (BLS102) with IPTG (1 mM) induction probed with anti-His antibody. Ponceau S-stained total protein gel for both samples serves as loading control. (C) Spot titer analysis to test the ability of *S. aureus glmR* (*glmR*^*Sa*^) to complement *B. subtilis* ∆*glmR* heterologously. The growth of WT (PY79), ∆*glmR* (SK35), *∆glmR glmR*^*Bs*^ (SK56), *∆glmR glmR*^*Sa*^ (SK27), and *glmR*^*Sa*^ (SK23) on LA and DS plates (containing 1 mM IPTG when indicated) were studied. Representative pictures of plates incubated overnight at 37 °C are shown.
(TIF)

S5 Fig Phosphomutants of GlmR are functionally similar to WT. (A) Spot titer assay of WT (PY79), ∆*glmR* (SK35), ∆*glmR glmR*^*+*^ (SK56), ∆*glmR glmR-T304A*^*+*^ (SK139), and ∆*glmR glmR-T304E*^*+*^ (SK140) on LA, LA + 1 mM IPTG, DS, and DS + 1 mM IPTG. (B) Micrographs of WT (PY79), ∆*glmR* (SK35), ∆*glmR glmR*^*+*^ (SK56), ∆*glmR glmR-T304A*^*+*^ (SK139), and ∆*glmR glmR-T304E*^*+*^ (SK140) with or without 1 mM IPTG induction. (C) Cell width quantifications of ∆*glmR glmR*^*+*^ (SK56), ∆*glmR glmR-T304A*^*+*^ (SK139), and ∆*glmR glmR-T304E*^*+*^ (SK140). (D) Colony area measurements relative to uninduced ∆*glmR glmR*^+^ (SK56), ∆*glmR glmR-T304A*^*+*^ (SK139), or ∆*glmR glmR-T304E*^*+*^ (SK140). Area measured via FIJI automatically using thresholding. One-way ANOVA with Tukey’s correction was used for interpreting statistical significance; * = p < 0.05, ** = p < 0.01, ns = p > 0.05.
(TIF)

S6 Fig Addendum to the working model. (A) As described in Fig 8, UDP-GlcNAc produced by GlmR and GlmU enzymes is consumed through the pathways involving MreB, MreBH, Mbl, and PBP1 in WT cells. In the absence of MreB, hyperactive PBP1 leads to abnormal cell bulging. This consequence is averted by either deletion of *pbp1* or overexpression of *glmR* (depicted in Fig 1B). When PBP1 is absent, alternative sigma factor SigI is activated which in turn upregulates *mreBH*. Therefore, the combined action of MreB and MreBH involved in cell width control leads to decreased cell width. In cells lacking *mbl*, UDP-GlcNAc utilization happens through both MreB and PBP1 pathways which result in twisted cell morphology. Thus, either deletion of *glmR* (lowers UDP GlcNAc level) or *pbp1* (increases MreBH activity) restores viability. (B) In cells lacking *glmR*, MreB pathway is weakened and PBP1 becomes hyperactivated. This leads to cell shape abnormality. Thus, either overexpression of *mreB* or deletion of *pbp1* results in cell morphology correction. Weak and strong UDP-GlcNAc consumption are represented with dashed and thicker arrows respectively.
(TIF)

S1 Video Aberrant positioning of cytokinetic machinery in the absence of GlmR. This Z-stack video shows *∆glmR* (SK35) cell undergoing abnormal septation. Red, FM 4–64 membrane dye. Movie created in FIJI.
(MP4)

S1 Raw Data Raw Data. (XLSX)
