# Supplementary material for: Post-translational modifications of GlmR integrate metabolic and stress signals to maintain cell envelope homeostasis in Bacillus subtilis
Source: PLoS Genet. 2026 Mar 30;22(3):e1012096. doi: 10.1371/journal.pgen.1012096 (PMC13046274; doi:10.1371/journal.pgen.1012096)
Supplement: S1 Text — Rescue of ΔglmR phenotypes by additional deletion of cdaA is likely due to polar effect. Deletion of gdpP, pgpH, or disA enhances the growth of ΔglmR mutant. Role of GlmR in cell morphogenesis. Potential role for potassium. Supplemental Methods. Table A: Strains used in this study. Table B: Primers used in this study. III. Supplemental. (DOCX) [file pgen.1012096.s001.docx]

Supporting Information

**Post-translational modifications of GlmR integrate metabolic and stress signals to maintain cell envelope homeostasis in *Bacillus subtilis***

Logan B. Suits^1,&^, Sebastian J. Khan^1,2,&^, Dipanwita Bhattacharya^1^, Silviya Dimitrova^1^, and Prahathees J. Eswara^1,3,*^

^1^Department of Molecular Biosciences, University of South Florida, Tampa, Florida, United States of America

^2^Present address: Cell and Developmental Biology Center, National Heart, Lung, and Blood Institute, National Institutes of Health, Bethesda, Maryland, United States of America

^3^Center for Antimicrobial Resistance, University of South Florida, Tampa, Florida, United States of America

^&^Contributed equally

*Prahathees J. Eswara: [eswara@usf.edu](mailto:eswara@usf.edu)

**I. Supplemental Results and Discussion**

*a) Rescue of ΔglmR phenotypes by additional deletion of cdaA is likely due to polar effect*

Our data reveals that the intracellular concentration of c-di-AMP is altered by GlmR (**Fig 4**). Therefore, we used fluorescence microscopy to investigate whether alteration in c-di-AMP concentration affects Δ*glmR* cell morphology. For this, we examined the cell morphologies of strains lacking *disA*, *cdaA*, and *cdaR* individually and in combination with *glmR* (**S2A Fig**). The cell morphologies of Δ*disA*, Δ*cdaA*, and Δ*cdaR* strains resembled WT. Therefore, depletion of intracellular c-di-AMP concentration alone does not affect cell morphology in our growth conditions. Conversely, Δ*disA* Δ*glmR* double-deletion mutant appeared similar to Δ*glmR*. Remarkably, in the Δ*cdaA* Δ*glmR* and Δ*cdaR* Δ*glmR* strains the typical abnormal Δ*glmR* cell morphology was completely abrogated. However, this result is counterintuitive as previous reports show only conditions that elevate c-di-AMP level, such as deletion of c-di-AMP phosphodiesterases alleviate the severity of Δ*glmR* phenotypes (1, 2). Therefore, we wondered if the reversal of Δ*glmR* phenotype could be due to the polar effect stemming from the antibiotic cassette mediated disruption of *cdaA* or *cdaR* (**S2B Fig**) - which introduces an additional promoter to drive downstream gene expression (3). More specifically, as GlmM is encoded from the highly conserved *cdaA*-*cdaR*-*glmM* operon (4), we suspected that the abrogation of Δ*glmR* phenotype by deletion of *cdaA* or *cdaR* could be explained by increased expression of *glmM* (**Fig 1A**). Overproduction of GlmM has been shown to rescue Δ*glmR* phenotypes previously (2, 5). Additionally, mutations that suppress Δ*glmR* phenotypes map to the *cdaA* locus and result in increased expression of *glmM* and/or *glmS* (**S2B Fig**; red asterisks) (2, 6). Therefore, we tested our prediction through two independent approaches - by either removing the antibiotic resistance marker from Δ*cdaA* (Δ*cdaA**) or by overexpressing *glmM* (*glmM*^+^) from an ectopic locus in the Δ*cdaA** background. As anticipated, unlike Δ*cdaA* Δ*glmR*, deletion of *glmR* in markerless Δ*cdaA** background did not fully correct the abnormal Δ*glmR* cell morphology (**S2C Fig**). On the contrary, increased expression of *glmM* in Δ*cdaA** Δ*glmR* background was sufficient to restore WT-like cell morphology.

We also employed the plate assay described in the main text to investigate the polar effect further (**Fig 5**). In **S3A Fig**, we show that the Δ*glmR* growth phenotypes (poor growth on LA and severe growth inhibition on DS) is corrected by the synthetic expression of inducible *glmS* from an ectopic locus. This effect was seen in MH medium as well (2). Next, we monitored the growth characteristics of Δ*cdaA* Δ*glmR* and Δ*cdaA** Δ*glmR* double mutants. Intriguingly, both strains grew better than *glmR* single deletion on LA and DS plates (**S3B Fig**). However, the growth of the former appears to be better than the latter. On DS, the growth of markerless version (Δ*cdaA** Δ*glmR*) was also significantly (>4-log) enhanced when compared to the Δ*glmR* strain. This is possibly due to the altered proximity to the native promoter which presumably leads to increased *glmM* expression (**S2B Fig**). We also ensured that both Δ*cdaA* and Δ*cdaA** do not exhibit any growth defect on their own (**S3C Fig**). Introduction of inducible *glmM* in Δ*cdaA** Δ*glmR* strain promoted growth on LA and DS even in the absence of inducer due to leaky expression. However, in the presence of inducer, the growth characteristics resembled that of WT on both plates (**S3B Fig**). Therefore, our results indicate that the reversal of Δ*glmR* cell morphology and growth phenotypes by *cdaA* deletion could be explained by the polar effect stemming from enhanced expression of *glmM*. Thus, researchers should exercise caution in interpreting results with knockout strains – even those with antibiotic resistance cassette removed.

*b) Deletion of gdpP, pgpH, or disA enhances the growth of ΔglmR mutant*

To investigate the possible effect of other c-di-AMP related proteins (**Fig 4A**), we tested the growth of the Δ*glmR* strain harboring an additional deletion of a phosphodiesterase gene of either GdpP or PgpH. First, we confirmed that all the individual knockout strains grow similar to WT on both media types (**S3C Fig**). On LA, we find that the *glmR* *gdpP* and *glmR* *pgpH* double deletion strains resemble Δ*glmR* (**S3D Fig**). However, on DS, we see significantly enhanced growth for ∆*glmR* ∆*gdpP* and mild improvement for ∆*glmR* ∆*pgpH*. This is consistent with previous findings (1, 2). It is to be noted that the growth of these strains are not quite similar to WT based on the opacity of the colonies on DS medium. We also investigated the growth of *glmR disA* double mutant (**S3E Fig**). The growth of strain harboring *disA* single deletion resembled WT control on both LA and DS. On LA, the growth phenotype of the ∆*disA* ∆*glmR* double deletion mutant appeared more similar to ∆*glmR*. Surprisingly, on DS, we find that this strain lacking DisA and GlmR grows nearly 4-log better than the ∆*glmR* single deletion strain. Thus, it appears that the absence of DisA is beneficial for the growth of ∆*glmR* cells in this specific experimental condition.

Why would both decrease (∆*disA*) and increase (∆*gdpP/*∆*pgpH*) of c-di-AMP levels promote the growth of cells lacking *glmR* (**S3D and S3E Fig**)? Our results shown in **Fig 4C** clarifies this, where we find that c-di-AMP level is in fact elevated in ∆*disA* ∆*glmR* compared to ∆*disA*. This increase in c-di-AMP level may be responsible for the better growth we see in ∆*disA* ∆*glmR*. Regarding phosphodiesterases, previous observations already demonstrated that disruption of *gdpP* or *pgpH* alleviates *glmR* deletion phenotypes (1, 2). Absence of GdpP or PgpH would also likely elevate the intracellular c-di-AMP concentration (7, 8). As we note that the level of c-di-AMP is reduced in ∆*glmR* cells (**S2E Fig**), it can be presumed that the increase in c-di-AMP level is critical for promoting the growth of ∆*glmR* cells specifically on DS (**S3E Fig**). It is to be noted that although the growth phenotype on plate is somewhat alleviated, the aberrant Δ*glmR* cell morphology is not corrected by *gdpP* or *pgpH* deletion (**S2D Fig**). High intracellular c-di-AMP concentration is known to assist with cell wall stress response (7, 9). However, the precise mechanism as to why deletion of *disA* or one of the phosphodiesterases in cells lacking *glmR* supports growth remains unclear. Our data suggests that GlmU function may become impaired on DS (**Fig 5C**). Thus, we can speculate that the deletion of *disA*/*gdpP*/*pgpH* in Δ*glmR* supports GlmU to remain at least partially active. Perhaps elevated c-di-AMP level may alter the intracellular magnesium concentration (10) and/or enhance the production or function of GlmS (**S3A Fig**) to subsequently bolster GlmU activity. Additional experiments are warranted to test these hypotheses.

*c) Role of GlmR in cell morphogenesis*

In our model, we propose that the balanced UDP-GlcNAc utilization by the MreB, Mbl, MreBH, and PBP1 pathways allows for cell width maintenance in WT (**S6A Fig**). There is evidence to suggest that MreB and MreBH are participating in the same pathway while Mbl works independently (11-13). It has been reported that the phenotypes of *mreB*/*mbl* overexpression are different (14). While MreB or MreBH overproduction is toxic, Mbl is not – thus hinting that Mbl function is possibly moderated in the presence of MreB/MreBH/PBP1 (thus the arrow thickness for Mbl remains unchanged in all the panels of **S6** and **8C, 8D, 8E Figs**). As per our model, in the absence of *mreB*, UDP-GlcNAc is consumed predominantly by PBP1 which causes cell bulging (**S6A Fig**). This effect can be relieved by *glmR* overexpression to elevate the supply of UDP-GlcNAc to support the functions of Mbl and MreBH (**Fig 1B**). Alternatively, deletion of *pbp1* also corrects the cell shape defects of *mreB* deletion strain through SigI-mediated upregulation of *mreBH* (15). Accordingly, deletion of *pbp1* alone would strongly favor the MreB/MreBH pathway and result in decreased cell diameter (**S6A Fig**). We suspect that the absence of Mbl promotes increased UDP-GlcNAc usage by PBP1 and MreB pathways giving rise to its unique spiral cell morphology (**S6A Fig**). As such, the rescue of Δ*mbl* viability can be achieved by additional deletion of *glmR* (13), as this would lead to less active MreB as well as decreased UDP-GlcNAc accumulation to limit PBP1 consumption.

Our results show that neither glucose nor magnesium supplementation fully restores the cell width of Δ*glmR* cells similar to WT (**Fig 3E**). We suspect that without GlmR, the tightly linked GlmR/MreB pathway becomes dysfunctional and UDP-GlcNAc made by GlmU (in the presence of glucose or magnesium) is primarily utilized by PBP1 and Mbl which supports rod shape maintenance. However, in the absence of GlmR, MreB is unable to fully participate. Thus, although rod shape is restored, cell width remains larger than WT in Δ*glmR* cells grown in the presence of glucose or magnesium.

*d) Potential role for potassium*

Differential inhibition of potassium channels by low vs high c-di-AMP concentrations has been noted (16). After osmotic upshift, c-di-AMP level drops immediately to favor intracellular potassium accumulation (and concomitant magnesium export) temporarily (10). It is known that potassium response to osmotic stress is more transient, and alternative compatible solutes such as glycine betaine and proline are subsequently favored (17). Therefore, perhaps increased potassium influx and subsequent dysregulated osmotic stress response activation could presumably be the source of Δ*glmR* toxicity. Intriguingly, polymerization of MreB is inhibited by high intracellular potassium concentration (18). Elevated levels of c-di-AMP would limit potassium influx and promote extrusion (19), which may therefore support MreB polymerization. As discussed above MreB is likely less active without GlmR – therefore, the predicted increase in c-di-AMP level in *glmR* *gdpP/pgpH* double mutants allows growth on plate (**S3D Fig**) even though is insufficient to correct the abnormal cell morphology of Δ*glmR* (**S2D Fig**). Intriguingly, high potassium concentration also dramatically alters the polymerization kinetics of the tubulin-like protein FtsZ, which determines the cell division site (20). Therefore, it is possible altered potassium level may contribute to the division site positioning defect that we observe in cells lacking *glmR* (**Fig 3B**). In addition to its role in osmotic stress response, potassium is also important for the maintenance of intracellular pH, membrane potential, ion homeostasis, and ribosome function to name a few (21-23). As such, additional experiments are warranted to elucidate the different pathways and possibilities.

**II. Supplemental Methods**

**Strain construction:**

*Bacillus subtilis* knockout strains were requested from the Bacillus Genetic Stock Center (BGSC). Chromosomal DNA of the strains harboring gene deletions were transformed into PY79 and confirmed using PCR. Plasmid pDR244 was used to generate markerless strains. Chromosomal insertion of genes of interest from integration vectors (pDG1662 or pDR111) were confirmed using standard protocol. *Escherichia coli* DH5a strain was used for plasmid maintenance. *E. coli* BL21-DE3 strain was used for protein purification.

**Plasmid construction:**

1. **c-di-AMP reporter**: The promoter harboring c-di-AMP riboswitch of *kimA* was amplified with oSK44/oSK45 from *B. subtilis* 168 chromosomal DNA and *gfp* with oSK46/oSK47. The resulting fragments were digested with EcoRI/NheI and NheI/BamHI, respectively, and ligated into pDG1662 digested with EcoRI/BamHI to create the plasmid pSK13.
2. **GlmR/mutant purification**: *glmR* was amplified from the PY79 chromosome using primers oSK48/oSK49; the resulting fragment was then digested with BamHI and NheI and ligated into pET28a (BamHI/NheI) to generate pSK14. The plasmid pSK14 was used for site-directed mutagenesis (QuikChange; Agilent) using primer pairs oSK60/oSK61, oSK56/oSK57, and oSK58/oSK59 to create versions of *glmR* harboring the R301A (pSK18), T304A (pSK17), and T304E (pDB04) mutations respectively.
3. ***glmR/mutant* complemention**: *glmR* was amplified from the PY79 genomic DNA using primers oSK16/oSK17; the resulting fragment was then digested with HindIII and SalI and ligated into pDR111 (HindIII/SalI), an IPTG-inducible vector for integration into the *amyE* locus in *B. subtilis* chromosome to generate pSK7. Similarly, *glmR* from pET28a plasmids harboring mutations R301A (pSK18), T304A (pSK17), and T304E (pDB04) were amplified using oSK16/oSK17 and cloned into pDR111 to generate pSK26, pSK24, and pSK25 respectively. pSK7 was amplified using the QuikChange kit (Agilent) with primer pairs oSK68/oSK69 and oSK70/oSK71 to introduce K296Q (pSK22) and K296R (pSK23) mutations respectively. To engineer the enzymatically-inactive GlmR mutant (D38A D39A), pSK7 was amplified with primer pairs oLS119/oLS120 to generate pLS59 using the QuikChange kit (Agilent). To generate *glmR-his6* and D38A D39A-his6, *glmR* from either pSK7 or pLS59 was amplified with oLS144/oLS145. The resulting fragments were digested with SalI/NheI and ligated into pDR111 resulting in plasmids pLS79 and pLS80, respectively. *Staphylococcus aureus* *glmR* (SAOUHSC_00788; *glmR^Sa^*) was amplified from SH1000 genomic DNA with oSK7/oSK8 primer pairs, and the resulting PCR products was digested with HindIII and SphI and cloned into pDR111 to make pSK5.
4. ***Other glm proteins***: *glmM* and *glmS* genes were amplified from PY79 genomic DNA using primer pairs oSK86/oSK87 and oLS69/oLS70 respectively. *glmM* fragment was digested with SalI/NheI and cloned into pDR111 to make oLS29. *glmS* PCR product was digested with XbaI/PstI and ligated into pBS2E-XylR-P*_xylA_* (ECE741; (24)) to generate oLS35.

**Table A: Strains used in this study**

| Strain | Genotype | Reference |
| --- | --- | --- |
| PY79 | Wildtype *B. subtilis* | (25) |
| RB176 | *glmR::erm* | BKE34760 (BGSC) 🡪 PY79 |
| SK23 | *amyE::P_hyperspank_-glmR^Sa^ spec* | pSK5 🡪 PY79 |
| SK27 | *glmR::erm amyE::P_hyperspank_- glmR^Sa^ spec* | pSK5 🡪 RB176 |
| SK29 | *glmR::erm*; *amyE::P_hyperspank_-glmR spec* | pSK7 🡪 PY79 |
| SK35 | *∆glmR* (markerless) | pDR244 🡪 RB176 |
| SK56 | *∆glmR*; *amyE::P_hyperspank_-glmR spec* | pSK7 🡪 SK35 |
| SK94 | *amyE::PkimA-gfp-cat* | pSK13 🡪 PY79 |
| SK96 | *cdaA::kan* | BKK01750 (BGSC) 🡪 PY79 |
| SK97 | *disA::kan* | BKK00880 (BGSC) 🡪 PY79 |
| SK98 | *gdpP::kan* | BKK40510 (BGSC) 🡪 PY79 |
| SK99 | *pgpH::kan* | BKK25330 (BGSC) 🡪 PY79 |
| SK101 | *cdaA::kan*; *glmR::erm* | SK96 🡪 RB176 |
| SK102 | *disA::kan*; *glmR::erm* | SK97 🡪 RB176 |
| SK103 | *gdpP::kan*; *glmR::erm* | SK98 🡪 RB176 |
| SK104 | *pgpH::kan*; *glmR::erm* | SK99 🡪 RB176 |
| SK107 | *glmR::erm*; *amyE::P_hyperspank_-glmR-K296Q spec* | pSK22 🡪 RB176 |
| SK108 | *glmR::erm*; *amyE::P_hyperspank_-glmR-K296R spec* | pSK23 🡪 RB176 |
| SK113 | *glmR::erm*; *amyE::P_kimA_-gfp cat* | SK94 🡪 RB176 |
| SK109 | *disA::kan*; *amyE::P_kimA_-gfp cat* | SK94 🡪 SK97 |
| SK110 | *cdaA::kan*; *amyE::P_kimA_-gfp cat* | SK94 🡪 SK96 |
| SK111 | *disA::kan*; *glmR::erm*; *amyE::P_kimA_-gfp cat* | SK94 🡪 SK102 |
| SK112 | *cdaA::kan*; *glmR::erm*; *amyE::P_kimA_-gfp cat* | SK94 🡪 SK101 |
| SK118 | *cdaR::kan*; *amyE::P_kimA_-gfp cat* | SK94 🡪 SK130 |
| SK119 | *glmR::erm*; *cdaR::kan*; *amyE::P_kimA_-gfp cat* | BKK01760 (BGSC) 🡪 SK113 |
| SK130 | *cdaR::kan* | BKK01760 (BGSC) 🡪 PY79 |
| SK131 | *cdaR::kan*; *glmR::erm* | SK130 🡪 RB176 |
| SK137 | *∆cdaA** (markerless) | pDR244 🡪 SK96 |
| SK138 | *∆cdaA**; *glmR::erm* | BKE34760 (BGSC) 🡪 SK137 |
| SK139 | *∆glmR*; *amyE::P_hyperspank_-glmR-T304A spec* | pSK24 🡪 SK35 |
| SK140 | *∆glmR*; *amyE::P_hyperspank_-glmR-T304E spec* | pSK25 🡪 SK35 |
| SK141 | *∆glmR*; *amyE::P_hyperspank_-glmR-R301A spec* | pSK26 🡪 SK35 |
| BLS67  BLS84 | *glmR::erm ∆cdaA* amyE::P_hyperspank_-glmM spec*  *∆glmR sacA::P_xyl_-glmS erm* | pLS29 🡪 SK138  pLS35 🡪 SK35 |
| BLS95 | *∆glmR*; *amyE::P_hyperspank_-glmR-D38A-D39A spec* | pLS59 🡪 SK35 |
| BLS101 | *∆glmR*; *amyE::P_hyperspank_-glmR-6his spec* | pLS79 🡪 SK35 |
| BLS102 | *∆glmR*; *amyE::P_hyperspank_-glmR-D38A-D39A-6his spec* | pLS80 🡪 SK35 |

**Table B: Primers used in this study**

| Primer | Sequence |
| --- | --- |
| oSK7 | AATAAAAGCTTACATAAGGAGGAACTACTATGAGACAAATAAAAGTTGTACTTATCG |
| oSK8 | AATAAGCATGCTTATTTACGTTTATCACTTGGTACGAAAGG |
| oSK16 | AATAA AAGCTT ACATAAGGAGGAACTACT ATGGGACAAAAGCCGAAAATC |
| oSK17 | AATAA GTCGAC TCATTCTTTCAGTAAATCAACAAGAAG |
| oSK44 | AATAA GAATTC CAGAATAAAACAGAGGCGATTTTAGCCTCTG |
| oSK45 | AATAA GCTAGC CATCGATGTCTTCCCCTTTTAATTTCTCATTTTTCAATTAAAACAATAAAACATCC |
| oSK46 | AATAA GCTAGC ATGAGTAAAGGAGAAGAACTTTTC |
| oSK47 | AATAA GGATCC TTATTTGTATAGTTCATCCATGCC |
| oSK48 | AATAAGCTAGCATGGGACAAAAGCCGAAAATC |
| oSK49 | AATAAGGATCCTCATTCTTTCAGTAAATCAACAAGAAG |
| oSK56 | GACGTAATACGTCACGATGCACATAAAGTGGCCTCTC |
| oSK57 | GAGAGGCCACTTTATGTGCATCGTGACGTATTACGTC |
| oSK58 | GACGTAATACGTCACGATGAACATAAAGTGGCCTCTCTTCTTG |
| oSK59 | CAAGAAGAGAGGCCACTTTATGTTCATCGTGACGTATTACGTC |
| oSK60 | CGTATAAAAATGACGTAATAGCTCACGATACACATAAAGTGGCC |
| oSK61 | GGCCACTTTATGTGTATCGTGAGCTATTACGTCATTTTTATACG |
| oSK68 | CAGAGATCAAATTGTAACGTATCAGAATGACGTAATACGTCACGATAC |
| oSK69 | GTATCGTGACGTATTACGTCATTCTGATACGTTACAATTTGATCTCTG |
| oSK70 | CAGAGATCAAATTGTAACGTATAGAAATGACGTAATACGTCACG |
| oSK71 | CGTGACGTATTACGTCATTTCTATACGTTACAATTTGATCTCTG |
| oSK86 | AATAAGTCGACACATAAGGAGGAACTACTATGGGCAAGTATTTTGGAACAG |
| oSK87 | AATAAGCTAGCTTACTCTAATCCCATTTCTGACC |
| oLS69 | AATAATCTAGAACATAAGGAGGAACTACTATGTGTGGAATCGTAGGTTATATCGG |
| oLS70 | AATAACTGCAGTTACTCCACAGTAACACTCTTCGC |
| oLS119 | CCAGAGCTCCCCCCAGCAGCGGCAACTGTTACAA |
| oLS120 | TTGTAACAGTTGCCGCTGCTGGGGGGAGCTCTGG |
| oLS144 | AATAA GTCGAC ACATAAGGAGGAACTACT ATGGGACAAAAGCCGAAAATCGC |
| oLS145 | AATAA GCTAGC TTAGTGATGGTGATGGTGATG TTCTTTCAGTAAATCAACAAGAAGAGAGGCC |

**IV. Supplemental References**

1. Gorke B, Foulquier E, Galinier A. YvcK of Bacillus subtilis is required for a normal cell shape and for growth on Krebs cycle intermediates and substrates of the pentose phosphate pathway. Microbiology (Reading). 2005;151(Pt 11):3777-91.

2. Patel V, Wu Q, Chandrangsu P, Helmann JD. A metabolic checkpoint protein GlmR is important for diverting carbon into peptidoglycan biosynthesis in Bacillus subtilis. PLoS Genet. 2018;14(9):e1007689.

3. Koo BM, Kritikos G, Farelli JD, Todor H, Tong K, Kimsey H, et al. Construction and Analysis of Two Genome-Scale Deletion Libraries for Bacillus subtilis. Cell Syst. 2017;4(3):291-305 e7.

4. Pham TH, Liang ZX, Marcellin E, Turner MS. Replenishing the cyclic-di-AMP pool: regulation of diadenylate cyclase activity in bacteria. Curr Genet. 2016;62(4):731-8.

5. Patel V, Black KA, Rhee KY, Helmann JD. Bacillus subtilis PgcA moonlights as a phosphoglucosamine mutase in support of peptidoglycan synthesis. PLoS Genet. 2019;15(10):e1008434.

6. Nicolas P, Mader U, Dervyn E, Rochat T, Leduc A, Pigeonneau N, et al. Condition-dependent transcriptome reveals high-level regulatory architecture in Bacillus subtilis. Science. 2012;335(6072):1103-6.

7. Luo Y, Helmann JD. Analysis of the role of Bacillus subtilis sigma(M) in beta-lactam resistance reveals an essential role for c-di-AMP in peptidoglycan homeostasis. Mol Microbiol. 2012;83(3):623-39.

8. Gundlach J, Mehne FM, Herzberg C, Kampf J, Valerius O, Kaever V, et al. An Essential Poison: Synthesis and Degradation of Cyclic Di-AMP in Bacillus subtilis. J Bacteriol. 2015;197(20):3265-74.

9. Corrigan RM, Abbott JC, Burhenne H, Kaever V, Grundling A. c-di-AMP is a new second messenger in Staphylococcus aureus with a role in controlling cell size and envelope stress. PLoS Pathog. 2011;7(9):e1002217.

10. Wendel BM, Pi H, Kruger L, Herzberg C, Stulke J, Helmann JD. A Central Role for Magnesium Homeostasis during Adaptation to Osmotic Stress. mBio. 2022;13(1):e0009222.

11. Dominguez-Cuevas P, Porcelli I, Daniel RA, Errington J. Differentiated roles for MreB-actin isologues and autolytic enzymes in Bacillus subtilis morphogenesis. Mol Microbiol. 2013;89(6):1084-98.

12. Sassine J, Sousa J, Lalk M, Daniel RA, Vollmer W. Cell morphology maintenance in Bacillus subtilis through balanced peptidoglycan synthesis and hydrolysis. Sci Rep. 2020;10(1):17910.

13. Koo BM, Todor H, Sun J, van Gestel J, Hawkins JS, Hearne CC, et al. Comprehensive double-mutant analysis of the Bacillus subtilis envelope using double-CRISPRi. bioRxiv. 2024.

14. Kawai Y, Asai K, Errington J. Partial functional redundancy of MreB isoforms, MreB, Mbl and MreBH, in cell morphogenesis of Bacillus subtilis. Mol Microbiol. 2009;73(4):719-31.

15. Patel Y, Zhao H, Helmann JD. A regulatory pathway that selectively up-regulates elongasome function in the absence of class A PBPs. Elife. 2020;9.

16. Rocha R, Jorge JMP, Teixeira-Duarte CM, Figueiredo-Costa IR, Cereija TB, Ferreira-Teixeira PF, et al. c-di-AMP determines the hierarchical organization of bacterial RCK proteins. Proc Natl Acad Sci U S A. 2024;121(18):e2318666121.

17. Whatmore AM, Chudek JA, Reed RH. The effects of osmotic upshock on the intracellular solute pools of Bacillus subtilis. J Gen Microbiol. 1990;136(12):2527-35.

18. Dersch S, Graumann PL. Adaptation of Bacillus subtilis MreB Filaments to Osmotic Stress Depends on Influx of Potassium Ions. Microorganisms. 2024;12(7).

19. Stulke J, Kruger L. Cyclic di-AMP Signaling in Bacteria. Annu Rev Microbiol. 2020;74:159-79.

20. Krol E, Scheffers DJ. FtsZ polymerization assays: simple protocols and considerations. J Vis Exp. 2013(81):e50844.

21. Stautz J, Hellmich Y, Fuss MF, Silberberg JM, Devlin JR, Stockbridge RB, et al. Molecular Mechanisms for Bacterial Potassium Homeostasis. J Mol Biol. 2021;433(16):166968.

22. Do EA, Gries CM. Beyond Homeostasis: Potassium and Pathogenesis during Bacterial Infections. Infect Immun. 2021;89(7):e0076620.

23. Helmann JD. Metals in Motion: Understanding Labile Metal Pools in Bacteria. Biochemistry. 2025;64(2):329-45.

24. Popp PF, Dotzler M, Radeck J, Bartels J, Mascher T. The Bacillus BioBrick Box 2.0: expanding the genetic toolbox for the standardized work with Bacillus subtilis. Sci Rep. 2017;7(1):15058.

25. Youngman P, Perkins JB, Losick R. Construction of a cloning site near one end of Tn917 into which foreign DNA may be inserted without affecting transposition in Bacillus subtilis or expression of the transposon-borne erm gene. Plasmid. 1984;12(1):1-9.

26. Elfmann C, Dumann V, van den Berg T, Stulke J. A new framework for SubtiWiki, the database for the model organism Bacillus subtilis. Nucleic Acids Res. 2025;53(D1):D864-D70.
