# Supplementary figures and images for: Post-translational modifications of GlmR integrate metabolic and stress signals to maintain cell envelope homeostasis in Bacillus subtilis
Source: PLoS Genet. 2026 Mar 30;22(3):e1012096. doi: 10.1371/journal.pgen.1012096 (PMC13046274; doi:10.1371/journal.pgen.1012096)

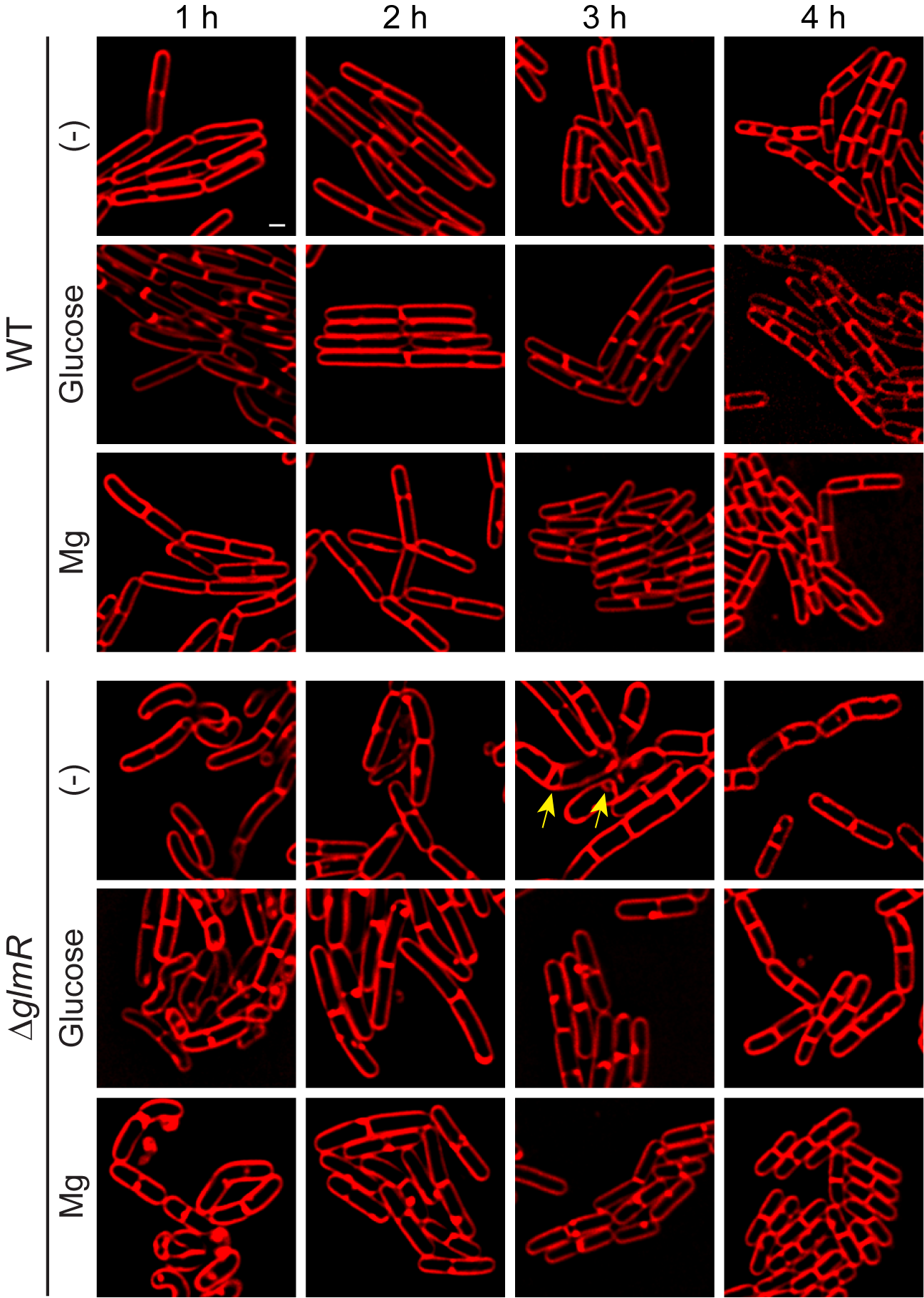

Supplement: S1 Fig — Representative micrographs of WT (PY79) and ∆glmR (RB176) strains grown in LB in the absence or presence of D-glucose (1%) or magnesium (25 mM MgCl2) supplementation, imaged hourly for four hours. Yellow arrows indicate examples of abnormal septation. (TIF) [file pgen.1012096.s002.tif]

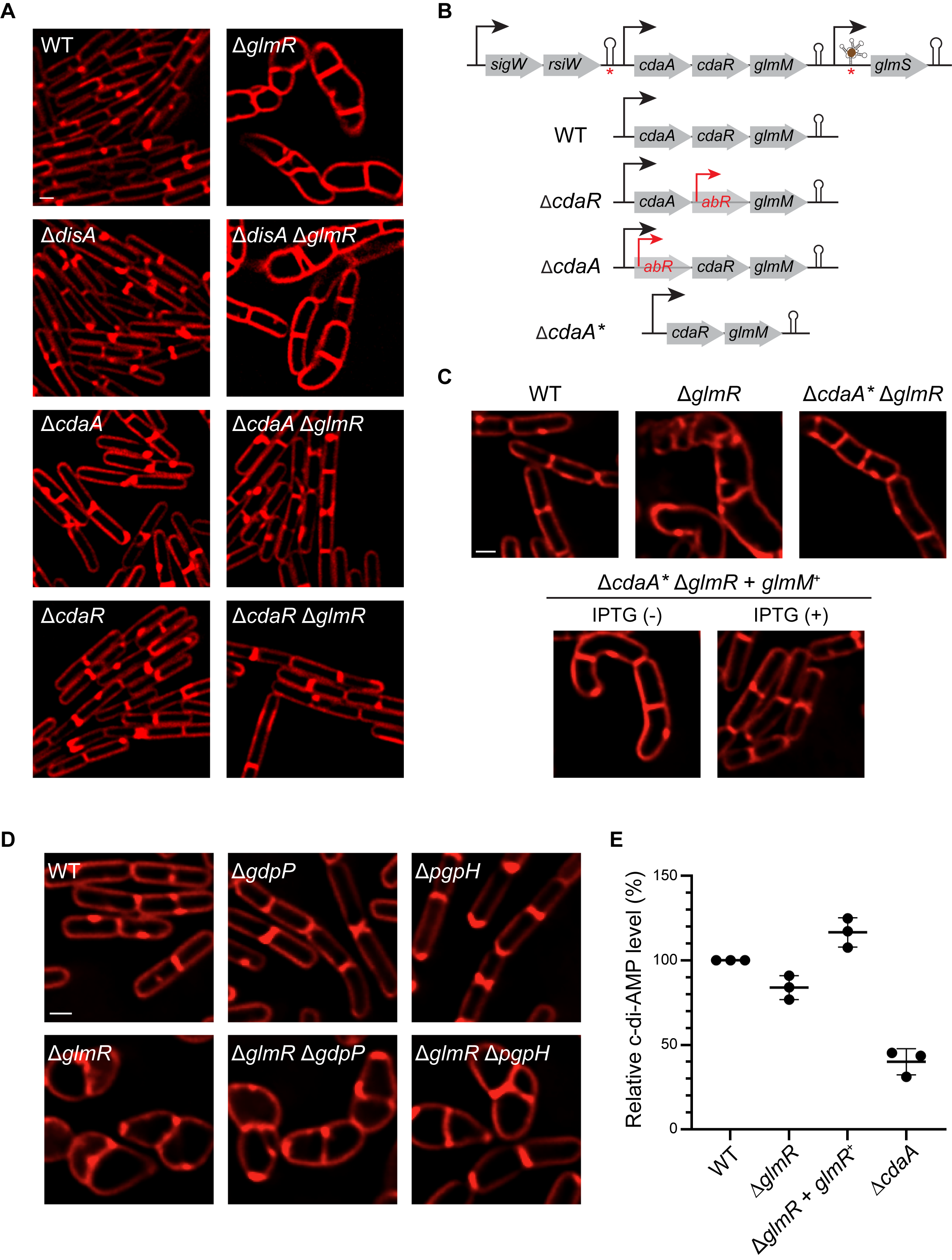

Supplement: S2 Fig — (A) Fluorescence micrographs of membrane-stained (FM 4–64, red): WT (PY79), ∆glmR (RB176), ∆disA (SK97), ∆disA ∆glmR (SK102), ∆cdaA (SK97), ∆cdaA ∆glmR (SK101) ∆cdaR (SK130), or ∆cdaR ∆glmR (SK131). Scale bar, 1 μm. (B) Genetic locus of cdaA-cdaR-glmM operon. Genes sigW-rsiW are located upstream of this operon while glmS is present immediately downstream in the B. subtilis genome. Red asterisks indicate the position of mutations commonly found in the ∆glmR suppressors that allow increased transcription of cdaA-cdaR-glmM genes and/or glmS. The terminator downstream of this cdaA operon is weaker (depicted with shorter symbol) resulting in read-through transcription of glmS. glmS has its own promoter followed by a riboswitch-ribozyme [71,72]. Replacement of cdaA or cdaR with an antibiotic resistance cassette introduces additional promoter (shown in red) and removal of the cassette replacing cdaA (∆cdaA*; markerless) brings glmM closer to its native promoter, thus potentially result in stronger expression. (C) Fluorescence micrographs of membrane-stained (FM 4–64, red): WT (PY79), ∆glmR (RB176), ∆glmR ∆cdaA* (SK138), and ∆glmR ∆cdaA* with inducible glmM+ (BLS67). When indicated, 0 and 1 mM IPTG was used in IPTG (-) and (+) conditions respectively. Scale bar, 1 μm. (D) Representative micrographs of WT (PY79), ∆glmR (RB176), ∆gdpP (SK98), ∆pgpH (SK99), ∆glmR ∆gdpP (SK103), and ∆glmR ∆pgpH (SK104). Red, FM 4–64 membrane stain. Scale bar, 1 µm. (E) ELISA-based intracellular c-di-AMP concentration estimation of WT (PY79), ∆glmR (SK35), ∆glmR complemented with inducible glmR (SK56), and ∆cdaA (SK130). Filled black circles represent technical replicates; error bars represent standard deviation. (TIF) [file pgen.1012096.s003.tif]

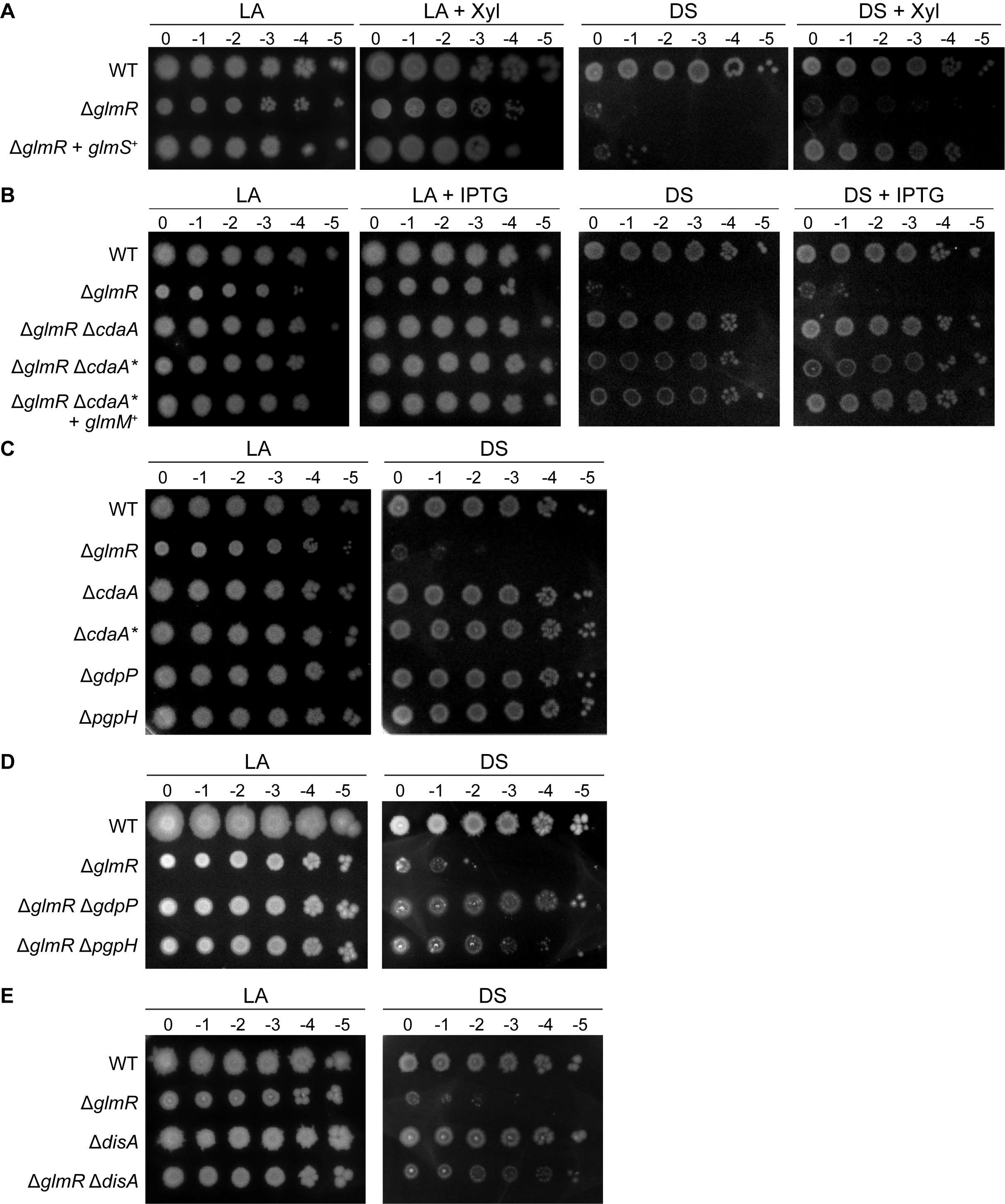

Supplement: S3 Fig — (A) Serial dilutions of WT (PY79), ∆glmR (SK35), ∆glmR glmS+ (BLS84) on LA, LA + 3% xylose, DS, or DS + 3% xylose. (B) Serial dilutions of WT (PY79), ∆glmR (RB176), ∆glmR ∆cdaA (SK101), ∆glmR ∆cdaA* (SK138), and ∆glmR ∆cdaA* glmM+ (BLS67) on LA, LA + 1 mM IPTG, DS, or DS + 1 mM IPTG. (C) Spot titer assay of WT (PY79), ∆glmR (SK35), ∆cdaA (SK96), ∆cdaA* (SK137), ∆gdpP (SK98), and ∆pgpH (SK99) on LA and DS. (D) Growth of serially-diluted culture aliquots of WT (PY79), ∆glmR (RB176), ∆glmR ∆gdpP (SK103), and ∆glmR ∆pgpH (SK104) on LA and DS plates. (E) Spot titer assay showing growth of WT (PY79), ∆glmR (RB176), ∆disA (SK97), and ∆glmR ∆disA (SK102) on LA and DS plates. (TIF) [file pgen.1012096.s004.tif]

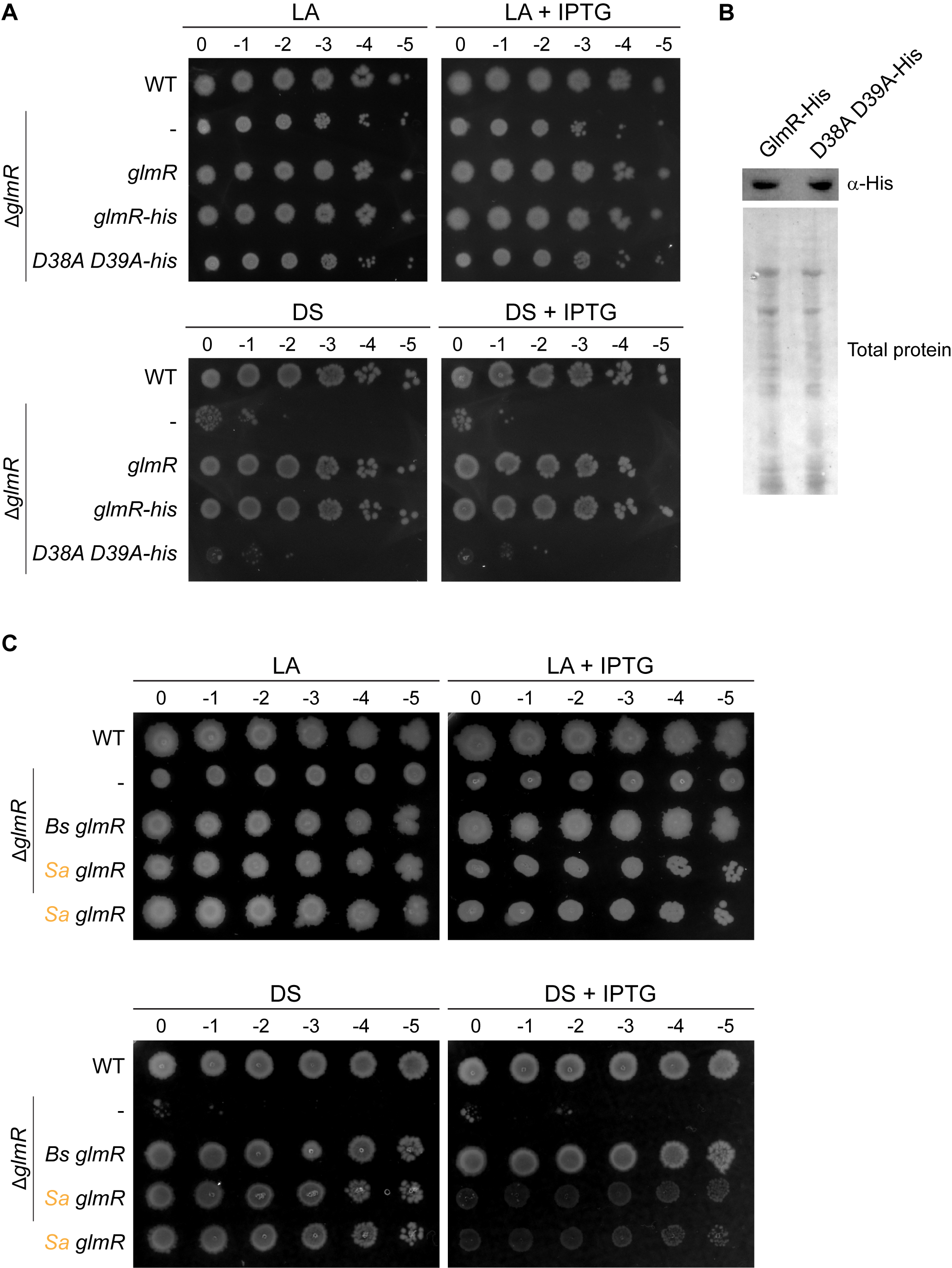

Supplement: S4 Fig — (A) Serial dilutions of WT (PY79), ∆glmR (SK35), ∆glmR glmR+ (SK56), ∆glmR glmR-6his+ (BLS101), and ∆glmR glmR-D38A-D39A-6his+ (BLS102) on LA, LA + 1 mM IPTG, DS, or DS + 1 mM IPTG. (B) Representative western blot of ∆glmR glmR-6his+ (BLS101) and ∆glmR glmR-D38A-D39A-6his+ (BLS102) with IPTG (1 mM) induction probed with anti-His antibody. Ponceau S-stained total protein gel for both samples serves as loading control. (C) Spot titer analysis to test the ability of S. aureus glmR (glmRSa) to complement B. subtilis ∆glmR heterologously. The growth of WT (PY79), ∆glmR (SK35), ∆glmR glmRBs (SK56), ∆glmR glmRSa (SK27), and glmRSa (SK23) on LA and DS plates (containing 1 mM IPTG when indicated) were studied. Representative pictures of plates incubated overnight at 37 °C are shown. (TIF) [file pgen.1012096.s005.tif]

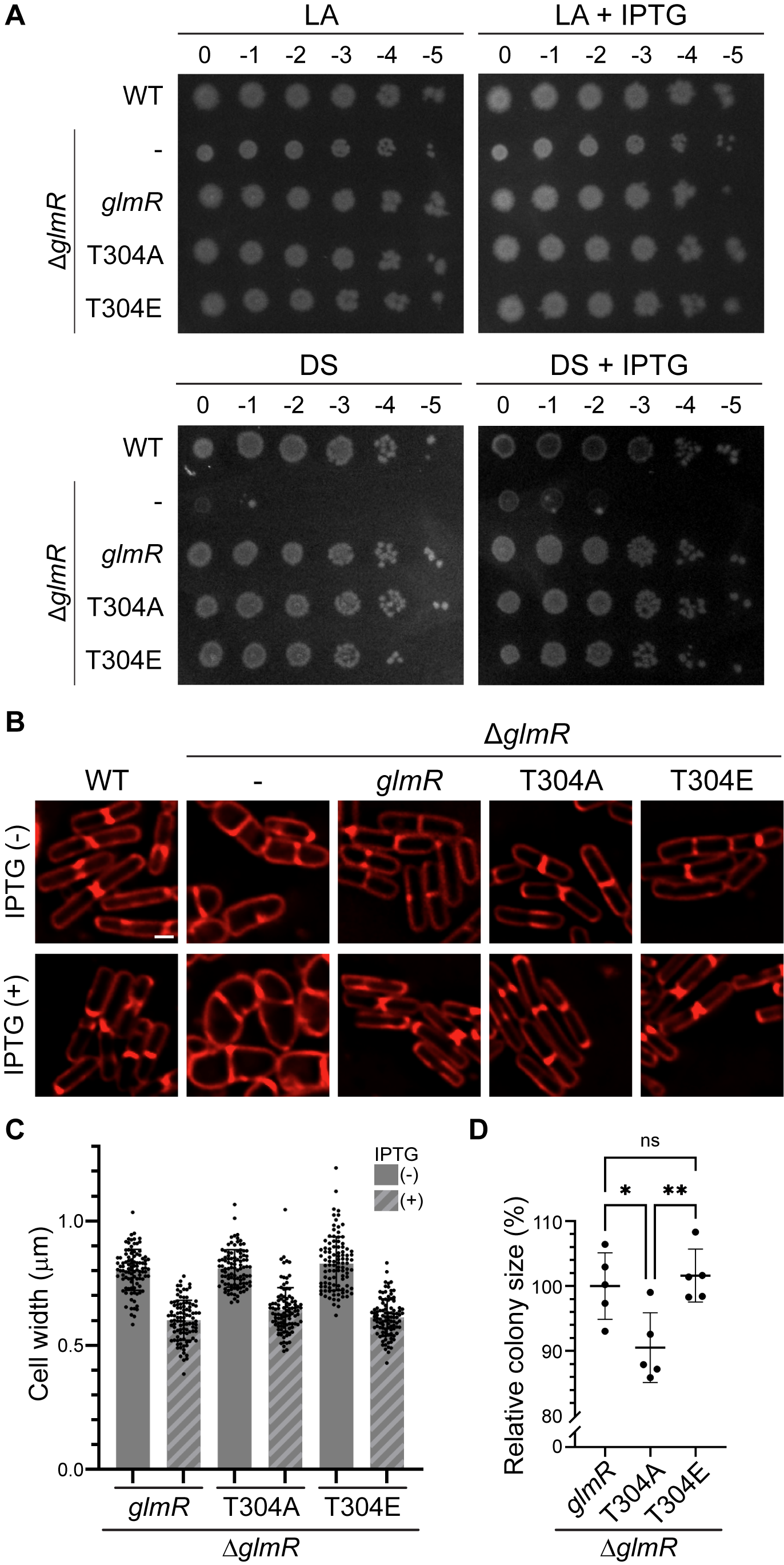

Supplement: S5 Fig — (A) Spot titer assay of WT (PY79), ∆glmR (SK35), ∆glmR glmR+ (SK56), ∆glmR glmR-T304A+ (SK139), and ∆glmR glmR-T304E+ (SK140) on LA, LA + 1 mM IPTG, DS, and DS + 1 mM IPTG. (B) Micrographs of WT (PY79), ∆glmR (SK35), ∆glmR glmR+ (SK56), ∆glmR glmR-T304A+ (SK139), and ∆glmR glmR-T304E+ (SK140) with or without 1 mM IPTG induction. (C) Cell width quantifications of ∆glmR glmR+ (SK56), ∆glmR glmR-T304A+ (SK139), and ∆glmR glmR-T304E+ (SK140). (D) Colony area measurements relative to uninduced ∆glmR glmR+ (SK56), ∆glmR glmR-T304A+ (SK139), or ∆glmR glmR-T304E+ (SK140). Area measured via FIJI automatically using thresholding. One-way ANOVA with Tukey’s correction was used for interpreting statistical significance; * = p < 0.05, ** = p < 0.01, ns = p > 0.05. (TIF) [file pgen.1012096.s006.tif]

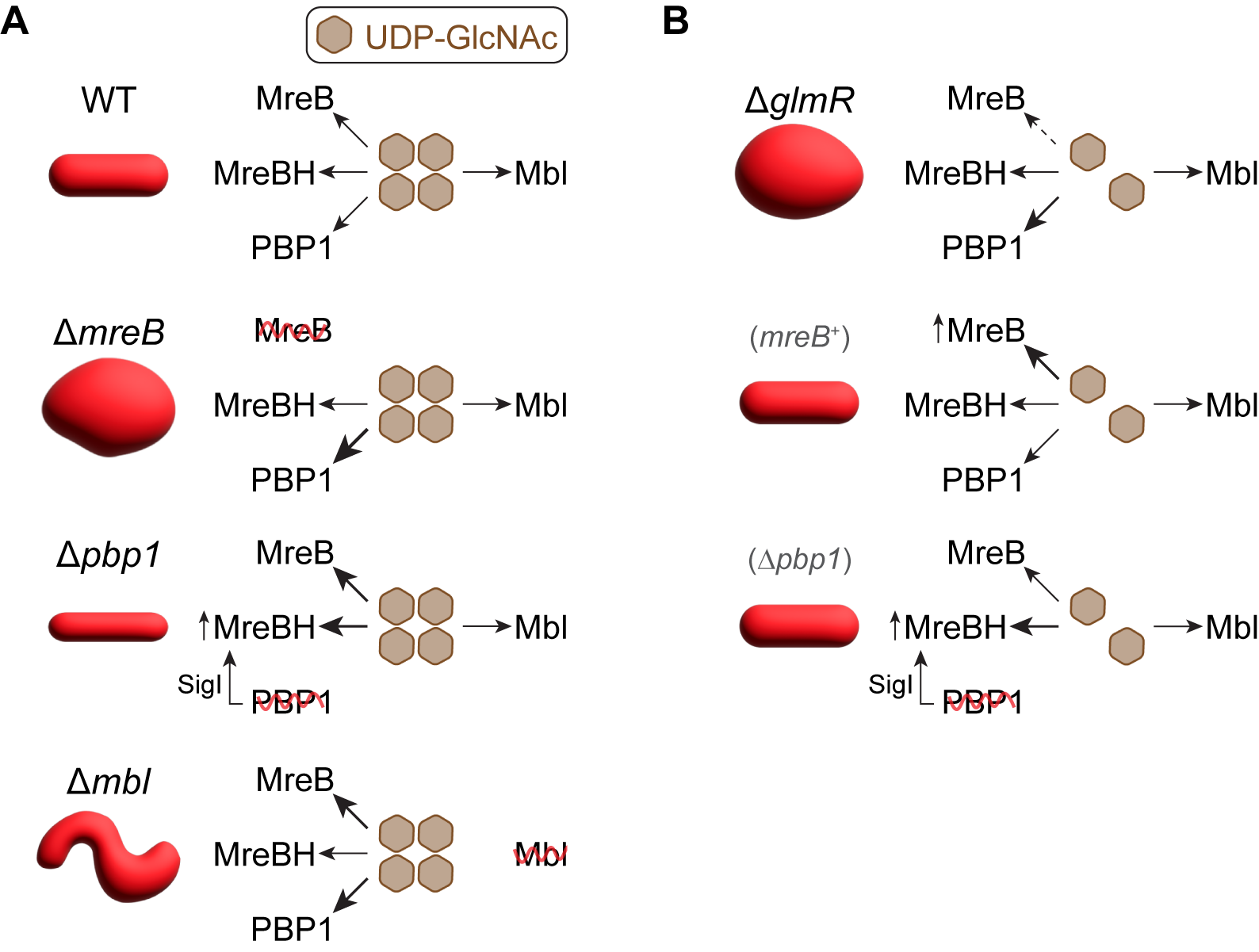

Supplement: S6 Fig — (A) As described in Fig 8, UDP-GlcNAc produced by GlmR and GlmU enzymes is consumed through the pathways involving MreB, MreBH, Mbl, and PBP1 in WT cells. In the absence of MreB, hyperactive PBP1 leads to abnormal cell bulging. This consequence is averted by either deletion of pbp1 or overexpression of glmR (depicted in Fig 1B). When PBP1 is absent, alternative sigma factor SigI is activated which in turn upregulates mreBH. Therefore, the combined action of MreB and MreBH involved in cell width control leads to decreased cell width. In cells lacking mbl, UDP-GlcNAc utilization happens through both MreB and PBP1 pathways which result in twisted cell morphology. Thus, either deletion of glmR (lowers UDP GlcNAc level) or pbp1 (increases MreBH activity) restores viability. (B) In cells lacking glmR, MreB pathway is weakened and PBP1 becomes hyperactivated. This leads to cell shape abnormality. Thus, either overexpression of mreB or deletion of pbp1 results in cell morphology correction. Weak and strong UDP-GlcNAc consumption are represented with dashed and thicker arrows respectively. (TIF) [file pgen.1012096.s007.tif]
